# Subsidising the spread of COVID-19: Evidence from the UK’S Eat-Out-to-Help-Out Scheme*

**DOI:** 10.1093/ej/ueab074

**Published:** 2021-10-26

**Authors:** Thiemo Fetzer

**Affiliations:** University of Warwick & CAGE, UK

## Abstract

This paper documents that a large-scale government subsidy aimed at encouraging people to eat out in restaurants in the wake of the first 2020 COVID-19 wave in the United Kingdom has had a significant causal impact on new cases, accelerating the subsequent second COVID-19 wave. The scheme subsidised 50% off the cost of food and non-alcoholic drinks for an unlimited number of visits in participating restaurants on Mondays–Wednesdays from 3–31 August 2020. Areas with higher take-up saw both a notable increase in new COVID-19 infection clusters within a week of the scheme starting and a deceleration in infections within two weeks of the program ending. Similarly, areas that exhibited notable rainfall during the prime lunch and dinner hours on the days the scheme was active record lower infection incidence—a pattern that is also measurable in mobility data—and non-detectable on days during which the discount was not available or for rainfall outside the core lunch and dinner hours.

The COVID-19 pandemic caused by the novel coronavirus (SARS-CoV) has left a significant mark on many economies. The hospitality sector is particularly vulnerable due to an unprecedented decline in tourism and leisure activities (Brinca *et al*., 2020; Carvalho *et al*., 2020; Dingel and Neiman, 2020). While lockdowns naturally limit the hospitality sectors’ ability to offer its goods and services, the changes in behaviour consumers adopt to avoid infections may result in the hospitality sector continuing to suffer from low demand (Baker *et al*., 2020; Mongey *et al*., 2020). As the changed consumption patterns are a direct result of the virus presence in many countries, economists have broadly suggested that aggressive testing and tracing schemes and the suppression of the virus’ spread may be the most cost-effective strategy to aid the economy (Brotherhood *et al*., 2020; DELVE, 2020; Kaplan *et al*., 2020). Nevertheless, some governments have leveraged fiscal policy to help the hospitality sector by stimulating demand: this paper studies to what extent a large-scale intervention in the UK, the so-called eat-out-to-help-out (henceforth, EOHO) scheme, had the unintended effect of furthering COVID-19 infections.

The EOHO scheme was conceived to shore up demand for the hospitality and restaurant sectors. It directly subsidised the cost of meals and non-alcoholic drinks by up to 50% across participating restaurants across the UK for meals served on Mondays–Wednesdays from 3–31 August 2020. The discount was capped at a maximum of GBP 10 per person, but there was no limit on how often individuals could benefit. Aggregate data suggest that 160 million meals were subsidised, costing the taxpayer GBP 849 million. Restaurant visits increased drastically on Monday to Wednesday, which usually see less traffic, even in a year-on-year comparison. Given the mounting evidence from epidemiology, which suggests that restaurants may be an important vector of COVID-19 transmission (see, e.g., Chang *et al*., 2020; Fisher *et al*., 2020; Hijnen *et al*., 2020; Lu *et al*., 2020; Marcus *et al*., 2020), this naturally raises the question whether the EOHO scheme contributed to COVID-19’s rapid spread.

This paper leverages spatially and temporarily granular data from England to make four observations. First, the EOHO scheme has led to a significant increase in restaurant visits over and above the levels in the previous year and to potentially shifting visits to the weekdays on which the discount was available. Second, areas that had more uptake of the scheme saw a notable increase in new COVID-19 infections detectable one week after the scheme launched. Third, the time patterns of the differential emergence of COVID-19 infections across areas with larger uptake closely track the time pattern of visits that the scheme appears to have induced when studying Google (2020) mobility data and aggregate data from restaurant booking sites. Fourth, we observe a notable decline in new infections in areas with higher take-up of the EOHO scheme around a week after the scheme ended. This pattern closely follows patterns in aggregate restaurant visit data, which saw a notable decline in restaurant visits after the scheme ended, suggesting that any positive economic impact was not sustained.

The difference-in-differences results suggest a robust link between the EOHO program and infections that is consistent with the state of epidemiological knowledge. Nevertheless, there may be concerns about reverse causality. I complement the above results with further reduced-form evidence that is, at least, indicative of the direction of causality. Using granular high-frequency hourly rainfall data, I show that areas that experienced notable rainfall *during lunch and dinner hours* on the weekdays during which the discount was available had fewer COVID-19 infections emerging relative to areas that saw little or no rain during these hours. These patterns are remarkably robust: rainfall during the same lunch and dinner hours on days in the week on which the discount was not available is uncorrelated with the emergence of new COVID-19 infection clusters. Similarly, rainfall that fell outside lunch and dinner hours on days during which the discount was available is uncorrelated with the subsequent emergence of COVID-19 infections. These patterns can only be detected during the four weeks when the scheme was active—but not in the four week windows before or after the scheme was active.

Naturally, rainfall may affect mobility in other ways and may have direct impacts on the spread of COVID-19. In order to at least partially allay these concerns, I again turn to daily district-level mobility data. Consistent with the above patterns on COVID-19 infections, I find that rainfall during lunch and dinner hours is associated with notably fewer restaurant visits. Furthermore, consistent with the results on infections, these effects are only present during the weekdays that the discount was available and for rain falling around the core lunch and dinner hours (but not for rainfall falling outside these hours or on weekdays on which the discount was not available). Similarly, these patterns are not detected in the four weeks prior or four weeks after the scheme ran. Lastly, the intra-day rainfall measures have no statistically discernible impact on mobility proxies capturing visits to grocery stores, transit or workplaces, suggesting that the patterns of reduced restaurant visits induced by rainfall around lunch and dinner hours are not confounding more general mobility changes. This is further indirect evidence suggesting that the scheme indeed *caused* an increase in infections.

The observed empirical results are robust to a host of further checks and exercises. First, it is noteworthy that the timing of the effects is very consistent with the EOHO scheme, both in terms of onset and offset, which is further confirmed by leveraging individual-level anonymised transaction data. Second, the results are robust to accounting for very demanding time effects that can capture various local policy shocks as well as account for the inherently non-linear local disease dynamics. Third, the results are robust to controlling non-parametrically for non-linear time trends in a large vector of factors that have been muted to drive the pandemic. Fourth, the results are not driven by any specific region or area. Fifth, the results are not an artefact of the specific choice of functional form or the precise measurement of the area-specific scheme take-up. This paper leverages detailed and granular restaurant-specific data from the government’s own public Github repository through which the central ‘restaurant finder’ application was run. This app was the primary go-to website that was used to help interested consumers identify participating restaurants within their neighbourhood.

The empirical estimates suggest that the EOHO scheme may be responsible for around 11% of all newly detected COVID-19 clusters emerging during August and into early September in the UK. These magnitudes map well to aggregate data from Public Health England that found that the share of infections traced back to restaurants increased from 5% at the start of the scheme to up to 20% by the end of the scheme, with the share subsequently rapidly declining again following a pattern that is very consistent with the other patterns identified. Given the dramatic rise of COVID-19 infections in late 2020 with nearly 80,000 deaths linked to COVID-19 since August 2020 and subsequent extended lockdowns and closures of the hospitality sector, this suggests that the EOHO scheme may have contributed to indirect economic and public health costs that vastly outstrip its short-term economic benefits. This paper is related to a rapidly growing literature studying the economic implications of the COVID-19 pandemic. The macroeconomic literature has put specific emphasis on understanding how to think of the optimal policy in the context of externalities in individual distancing decisions and socially optimal lockdowns. Guerrieri *et al*. (2020) studied optimal fiscal or monetary policy in the context of COVID-19. They showed that an optimal policy response involves shutting down ‘contact intensive’ sectors and providing full compensation to employees. This paper also relates to notable work that is being conducted to track the economic implications of the pandemic in real time across a host of margins, such as inequality (Adams-Prassl *et al*., 2020b,c; Benzeval *et al*., 2020; Blundell *et al*., 2020), gender-differentiated effects (Alon *et al*., 2020) and across sectors and social economic groups (Coibion *et al*., 2020b; Mongey *et al*., 2020). There is also a growing literature that studies the impacts and implications of various conventional and unconventional fiscal countermeasures (see D’Acunto *et al*., 2018; Bayer *et al*., 2020; Coibion *et al*., 2020a,b; Kaplan *et al*., 2020; D’Acunto *et al*., 2021, to name a few).

This paper puts an emphasis on a specific fiscal countermeasure that, even at the time of announcement, has been criticised by epidemiologists and economists for its ‘backfire potential’ given the known risks of infection in restaurant settings.

In the broader literature, this paper is related to the strand of work that speaks to the complexity of economic policy making in the wake of a pandemic in a world with both economic externalities and health externalities. Targeted fiscal interventions may be optimal if they reduce both the negative economic impacts of the pandemic and, at the same time, put in check the underlying health externalities that certain types of economic behaviour may bring about. Most countries opted for a broad set of measures to prevent sectors from making drastic adjustments to its workforce through the expansion of furlough schemes (see Adams-Prassl *et al*., 2020a for work on the UK scheme), targeted financial and liquidity support to companies, as well as broad demand stabilising initiatives such as those implemented through the CARES Act in the United States (Coibion *et al*., 2020b) or through measures to temporarily lower the VAT.

The UK’s policy response shared many of the broader features of most fiscal interventions, yet the EOHO scheme stands out internationally. The intervention not only reversed a lockdown that ordered in-dining restaurants to shut (as, e.g., studied in Glaeser *et al*., 2020), but was rather designed to actively increase demand for the hospitality sector. Given a broad set of epidemiological work that suggests that the health externalities associated with hospitality-sector-related economic activity may be particularly high (see, e.g., Chang *et al*., 2020; Fisher *et al*., 2020; Hijnen *et al*., 2020; Lu *et al*., 2020), the overall soundness of the scheme stands in question.

This paper proceeds as follows. Section 1 presents the policy context and the underlying data leveraged in this paper. Section 2 presents the empirical approach, while Section 3 presents and discusses the results. Section 4 concludes.

## 1. Context and Data

### 1.1. Measuring COVID-19 spread in the UK

I leverage data from the UK’s official COVID-19 reporting dashboard available at https://coronavirus.data.gov.uk/. This provides data on weekly COVID-19 case counts at the Middle Layer Super Output Area (MSOA) level.^1^ MSOAs have, on average, 8,288 residents and at least 5,000 residents as per the 2011 census. Across the study area of England there are 6,791 MSOAs. For confidentiality protection, the data suppress counts that are less than or equal to 2. Individual cases get allocated to individual MSOAs based on their residence address. Furthermore, cases get allocated to weeks based on the date on which individuals take the COVID-19 test—which, due to processing delays, can be different from the date that a test result is reported (see Fetzer and Graeber, 2020 for a related paper).

The resulting dataset at the MSOA level is a balanced panel across 6,791 English MSOAs by calendar week from calendar week 5 to calendar week 40. For ease of interpretation, the primary dependent variable this paper studies is whether an MSOA reported more than two new COVID-19 cases in any given week—though all results are robust and provide similar quantitative results when using the continuous case count measures. While the dataset stretches all the way to the beginning of the year, I focus on a shorter time window around the time during which the EOHO scheme was running. All results are robust to including data from early in 2020.

Online Appendix Figure A1 highlights the rapid spread of COVID-19: in the week prior to the launch of the EOHO scheme, just 7% of English MSOAs reported a new local outbreak with more than two new cases; only four weeks later, as the scheme ended, 30.6% of MSOAs reported local outbreaks. We assess to what extent the government-operated EOHO scheme may have contributed to the spread.

### 1.2. Eat-Out-To-Help-Out Scheme

The hospitality sector in the UK, as elsewhere, took a significant hit due to COVID-19. The government implemented a demand-inducing subsidy that was dubbed the ‘eat-out-to-help-out’ scheme. Under the scheme, the government covered 50% of the cost of food and non-alcoholic drinks consumed in participating restaurants UK wide. The scheme ran from 3 August (calendar week 32) to 31 August 2020 (calendar week 36), but was only available on Mondays–Wednesdays. The discount was capped at a maximum of GBP 10 per person with no limit on how often individuals could benefit. Eligible businesses had to register with the UK’s Tax Authority, Her Majesty’s Revenue and Customs (HMRC). Once registered, businesses could offer the discount to customers and claim the money back from HMRC.

**Fig. 1.**
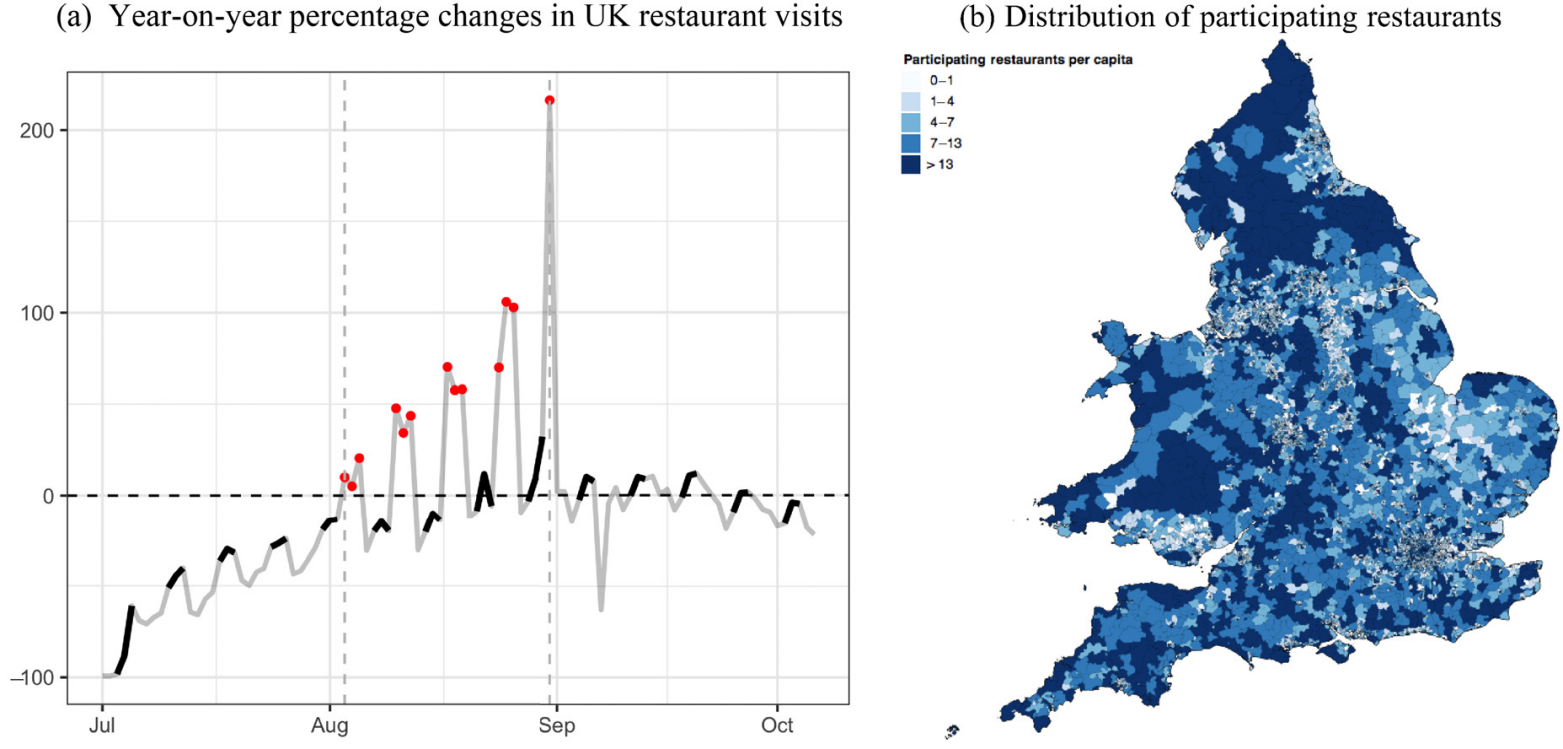
Restaurant Visits over Time and Spatial Distribution of Participating Restaurants across England and Wales. *Notes:* Panel (a) plots year-over-year proportional changes in seated diners at a sample of restaurants on the OpenTable network across all channels: online reservations, phone reservations and walk-ins across the UK in percentages. The vertical lines indicate the start and end dates of the EOHO scheme. The individual dates when the EOHO subsidy was available across participating restaurants in the UK are marked as circles. Dark shaded days mark Fridays, Saturdays and Sundays. August 31 was the last so-called Summer Bank Holiday that marks a public holiday. The date of this bank holiday changes each year, with the corresponding holiday the previous year being 26 August 2019. This results in a notable inflation in restaurant visits in the year-on-year comparison for these two dates. Panel (b) plots the distribution of the number of participating restaurants per 10,000 residents at the MSOA level.

Official subnational statistics were released at the end of January 2021. These statistics suggest that at least 59,981 businesses have registered for the scheme and discounts for more than 106 million meals were claimed. The average claim was GBP 5.74, just over half the GBP 10 maximum per person. Figure 1(a) highlights that the program did have a notable temporary impact on restaurant visits when comparing year-on-year changes from UK time series from the booking service OpenTable. During days that the scheme was available, restaurant visits increased between 10%–200% year-on-year. The data also suggest that the scheme may have shifted restaurant visits from the weekend to weekdays on which the discount was available and that the increased restaurant activity was of a temporary nature.

#### 1.2.1. Restaurant registrations

I leverage data from HMRC’s restaurant registration database, which was made available for developers through Github. This repository was used by HMRC to develop the government's EOHO restaurant finder app to help consumers find participating restaurants within five miles of any postcode.^2^ Registrations were open during the full program duration. Online Appendix Figure A3 presents the time series of registered restaurant businesses. While the vast majority of restaurant businesses were individual restaurants, several large chains also participated. These are not all represented in the Github data. Using the list of chains that participated provided by HMRC, together with point-of-interest data from the UK’s Ordnance Survey, I add all premises linked to restaurant chains that participated. The results are very similar, excluding data pertaining to restaurant chains.^3^

We construct cross-sectional measures capturing the intensity of the program’s use across MSOAs to study whether the program is associated with an acceleration in the spread of the pandemic. Figure 1(b) displays the distribution of the number of participating restaurants per 10,000 residents at the end of the program.

#### 1.2.2. Official local EOHO statistics

A second way of measuring uptake is to leverage data that were published at the level of the constituency. The data provide information on the number of participating restaurants by constituency; the number of meals claimed; the amount of money disbursed to participating restaurants and the average value per claim. The data were released in late January 2021. To leverage the other data on take-up, I merge the coarser 533 constituencies with the granular MSOAs. The reported constituency figures on the number of meals claimed are broken down to the MSOA level using the number of participating restaurants in each MSOA from the restaurant finder app as a weight. This gives us a measure of the number of meals claimed as an additional inferred measure of uptake. All results obtained here are robust to using the restaurant-only data that come directly from HMRC’s Github’s repository representing the universe of participating restaurants.

### 1.3. Rainfall Data and Other Data

#### 1.3.1. Rainfall data

I use data from the Global Satellite Mapping of Precipitation project that provides the hourly rain rate with a 0.1 × 0.1 degree resolution (around 10 × 10 km at the equator) provided in Okamoto *et al*. (2005). To construct the rainfall measures at the MSOA level, I obtain the hourly images and map the grid cell to the centroid for each MSOA. To construct the rainfall during the lunch and dinner hours, I sum up the hourly rainfall rates on each day for lunch hours from 11:00–14:00 and dinner hours from 17:00–21:00. Focusing on rainfall outside these hours provides a natural placebo. For ease of interpretation, the primary rainfall measure I leverage is coded as a binary indicator capturing whether an area’s rainfall was in the upper decile of the empirical distribution. As the infection data at the local level are provided only at the weekly level, I aggregate the rainfall occurring on weekdays during which the EOHO discount was available (Mondays–Wednesdays) as well as for the rest of the week. For the mobility exercise, I can leverage the daily rainfall data directly.

#### 1.3.2. Google mobility data

To understand to what extent the EOHO scheme changes or affects local patterns directly, I also leverage daily data from the Google Mobility indices. The data are provided broadly speaking at the local authority districts level. I use the daily-level data to measure the impact of the EOHO scheme on mobility within districts over time to provide corroborating evidence.

## 2. Empirical Strategy

### 2.1. Difference-in-Differences Analysis

I use several simple difference-in-differences strategies to exploit cross-sectional variation across MSOAs in the take-up of the EOHO scheme $E_{i}$. I estimate

(1) $$\begin{equation*}
y_{i,t} = \eta _i + \gamma _{l(i),t} + \eta \times \textit{Post}_t \times E_{i} + \beta ^{\prime } X_{i,t} + \epsilon _d ,
\end{equation*}$$

where $y_{i, t}$ denotes a measure of COVID-19 spread. The primary focus of this paper is to measure the emergence of new COVID-19 infection clusters defined as more than two new cases per MSOA per week. The results are not sensitive to the choice of the dependent variable or the functional form, as will be shown.

The regression controls for district or MSOA fixed effects, $\eta _i$, as well as a set of time fixed effects, $\gamma _{l(i),t}$. I explore a range of different time fixed effects; for example, I can control for district by time fixed effects or Westminster constituency by time fixed effects. The potential for local policy shocks (local policy is formulated at the district level) and area-specific non-linear growth in COVID-19 makes such flexible time effects particularly suitable. Here $E_{i}$ is a measure of an area’s exposure to the EOHO scheme. I work with two primary measures that capture either the number of restaurant establishments within an MSOA that participate in the EOHO scheme or an imputed measure of the number of meals covered under the scheme. In the preferred specification these are measured in per capita terms, though, given that MSOAs have—by construction—similar population, the results are virtually unaffected by this choice. The above specification measures how areas with higher exposure to EOHO, measured by $\ E_i$, saw a differential increase in COVID-19 cases following the program's start, i.e. when $\ Post_t\,=\,1$. Naturally, we also can estimate a more flexible econometric specification that allows us to explore to what extent there are common trends in infection outcomes before the scheme started, e.g., by estimating

$$\begin{equation*}
y_{i,t} = \eta _i + \gamma _{l(i),t} + \sum _{t} \eta _t \times \mathbb {1}(\textit{Week}=t) \times E_{i} + \beta ^{\prime } X_{i,t} + \epsilon _d .
\end{equation*}$$

What is important in the above specification is that we can not only explore how the program may have led to an increase in infection *from the onset*, but we can also study to what extent infection dynamics slow down as the scheme ends. Throughout the paper, we cluster SEs at the level of the local authority district.

#### 2.2.1. Control variables

In addition to conducting various robustness checks, we can control for a host of potential time-varying factors measured in $X_{i,t}$ that may drive the spread of the disease independently from the EOHO scheme.

### 2.2. Exploiting Time Variation in (Likely) Restaurant Visits

At the time this paper was written, time-varying subnational measures of EOHO uptake were not available. To allay concerns about reverse causality, I also adopt a reduced-form approach that exploits time variation in inclement daily weather across England around the lunch and dinner hours. Specifically, I focus on the period during which the discount was available, exploiting time variation within and between MSOAs in whether an area saw notable rainfall during the primary lunch and dinner hours when individuals may have conceivably taken advantage of the discount to visit restaurants. I estimate

(2) $$\begin{equation*}
y_{i,t} = \eta _i + \gamma _{l(i),t} + \xi \times R_{i,t} + \epsilon _d ,
\end{equation*}$$

where, again, $y_{i,t}$ is a dummy variable that measures whether a new COVID-19 infection cluster was identified in an area in a calendar week. The timing is not particularly sharp, but epidemiological estimates suggest that 97% of non-asymptomatic patients develop symptoms within 8.2 to 15.6 days of infection (Lauer *et al*., 2020). At least 50% of symptomatic cases report symptoms within two to five days of infection (see, e.g., Chun *et al*., 2020; Qin *et al*., 2020); it is thus not unreasonable for COVID infections occurring at the start of the week to be detected among tests conducted later in the same week (infections are measured based on the date on which a test was taken). I will show that the results are not an artefact of the precise choice of timing.

The above regression controls for area-specific time fixed effects, again, to account for non-linear growth and potential confounding policy shocks. I construct a set of different rainfall measures $R_{i,t}$ that measure the amount of rainfall from 11:00–14:00, proxying lunch hours, and from 17:00–21:00, proxying dinner hours. These measures are constructed for each day. I also construct a rainfall measure outside these time windows. This allows a host of placebo exercises that will be further supported by the mobility analysis described next.

### 2.3. Supporting Mobility analysis

I leverage data from Google Mobility indices to measure times spent in retail, restaurant, parks, workplaces or at home at the local authority district. This allows for two additional exercises that can corroborate the reduced-form evidence. First, at the week level I show that mobility patterns proxying time spent and visits to restaurants significantly responded to the roll out of the scheme. I estimate variations of specification (1) at the district level, documenting how mobility proxies proxying restaurant visits changed on EOHO days compared to EOHO weekdays both before and after the scheme started and subsequently after it ended.

Second, I study the daily data for just the time period during which the scheme was active, exploiting the daily weather variation, now at the district level, as discussed above. Specifically, I explore variations of specification (2) to study to what extent restaurant visit mobility proxies appear to decrease on EOHO weekdays in districts that experienced some notable rainfall. Again, this exercise allows for a host of additional placebo exercises, the results of which closely track the patterns identified from the infection data.

## 3. Results and Discussion

### 3.1. Difference-in-Differences Analysis

#### 3.1.1. The EOHO scheme increased restaurant visits

We begin by studying the Google mobility data to explore to what extent the scheme has led to more restaurant visits—and to what extent the patterns change after the program ended. Figure 1(a) suggested that, nationally, the EOHO scheme may have led to a notable increase in restaurant visits year-on-year and a shift in visits within the week. We make three observations. First, while restaurant visits had been recovering relative to the previous year in July, there are notably higher restaurant visits on days during which the scheme was available—the increases suggest that restaurant visits year-on-year are between 10% to more than 200% higher. Second, the patterns suggest that, prior to the scheme being active, there is a notable increase in visits during weekend days within a week—this pattern seems to disappear during the weeks that the EOHO scheme was available, which may suggest that the scheme may have led to the shifting of planned visits within the week to days during which the discount was available. Third, there is a notable downward trend in restaurant visits after the scheme ended.

While the Google mobility measures do not provide a sharp measure that covers just restaurants, the results presented in Online Appendix Figure A4 nevertheless suggest that the scheme had a signifiant and timely effect, increasing mobility in the retail and recreation category, which includes restaurants and cafes. On Mondays to Wednesdays, during which the program was active, the mobility score increased drastically by around 6 percentage points. Relative to the mean, this is an increase of around 22%.^4^ The results further suggest that, as the scheme ended on August 31, time spent in restaurants and cafes significantly dropped again from week 36 and did not recover. These observations map well to the patterns observed from the aggregate restaurant booking data.^5^

#### 3.1.2. Impact of the EOHO scheme on infections

We next turn to presenting results from the difference-in-differences analysis. The results are presented in Table 1. As the dependent variable (DV) in this exercise we use the binary indicator that measures whether there was a new COVID-19 infection cluster comprising more than two new cases detected within a given calendar week. The sample period here covers calendar weeks 24 to 36. Across the different panels in the regression table, I explore different ways of measuring the exposure of an area to the EOHO scheme. In panel A, I measure the exposure as the log number of EOHO-covered meals consumed in an area or the log number of participating restaurant establishments in an area, each normalised by an area’s population. For ease of interpretation, the measures are normalised to have unit SD. Across columns, I explore different levels of time fixed effects, moving from coarser NUTS2-region-by-week fixed effects to much more granular local-authority-district-by-week fixed effects.

**Table 1.** Impact of the EOHO Scheme on the Emergence of Local Infection Clusters.

DV: any new COVID-19 cluster	(1)	(2)	(3)	(4)	(5)	(6)
*Panel A: EOHO exposure measured in logs normalised by the population*						
Post $\times$ log(EOHO-covered meals per capita)	0.007$^{***}$	0.009$^{***}$	0.009$^{***}$			
	(0.003)	(0.003)	(0.003)			
Post $\times$ log(EOHO restaurants per capita)				0.008$^{***}$	0.009$^{***}$	0.011$^{***}$
				(0.003)	(0.003)	(0.003)
Mean DV	0.096	0.096	0.096	0.096	0.096	0.096
Observations	88,283	88,283	88,283	88,283	88,283	88,283
MSOAs	6,791	6,791	6,791	6,791	6,791	6,791
Additional controls	390	1,209	4,121	390	1,209	4,121
Clusters	317	317	317	317	317	317
*Panel B: EOHO exposure measured in* $\textrm{log}\, +$ 1						
Post $\times$ log(EOHO meals)	0.017$^{***}$	0.017$^{***}$	0.016$^{***}$			
	(0.004)	(0.003)	(0.003)			
Post $\times$ log(EOHO restaurants)				0.014$^{***}$	0.016$^{***}$	0.017$^{***}$
				(0.003)	(0.003)	(0.003)
Mean DV	0.097	0.097	0.097	0.096	0.096	0.096
Observations	79,547	79,547	79,547	88,283	88,283	88,283
MSOAs	6,119	6,119	6,119	6,791	6,791	6,791
Additional controls	390	1,209	4,121	390	1,209	4,121
Clusters	317	317	317	317	317	317
*Panel C: EOHO exposure measured in levels*						
Post $\times$ EOHO-covered meals	0.015$^{***}$	0.015$^{***}$	0.014$^{***}$			
	(0.004)	(0.003)	(0.004)			
Post $\times$ EOHO restaurants				0.011$^{**}$	0.011$^{**}$	0.012$^{**}$
				(0.005)	(0.005)	(0.005)
Mean DV	0.096	0.096	0.096	0.096	0.096	0.096
Observations	88,283	88,283	88,283	88,283	88,283	88,283
MSOAs	6,791	6,791	6,791	6,791	6,791	6,791
Additional controls	390	1,209	4,121	390	1,209	4,121
Clusters	317	317	317	317	317	317
**Area by week FE**s	NUTS2	NUTS3	LAD	NUTS2	NUTS3	LAD

*Notes:* This table presents difference-in-differences regression estimates of the impact of the EOHO scheme at the MSOA level on the emergence of new COVID-19 infection clusters across the thirteen calendar weeks (24 to 36). A COVID-19 infection cluster is defined as a week in which there were strictly more than two new COVID-19 infections detected in tests taken during the week. All regressions control for MSOA-level fixed effects. The regression also control for week fixed effects specific to each NUTS2, NUTS3 or local authority. SEs are clustered at the district level with $^{***} p\lt 0.01$, $^{**} p\lt 0.05$, $^{*} p\lt 0.1$.

The results suggest that there is a notable positive and precisely estimated impact: areas that have higher uptake to the EOHO discount see a notably higher incidence of infections during the weeks that the program ran. In panels B and C I show that it is immaterial how we measure the exposure to the scheme in this difference-in-differences exercise. Overall, the estimates across columns and panels suggest that a one SD higher exposure to the EOHO scheme increased the incidence of new infection clusters by, on average, between 0.008 to 0.017 percentage points. Relative to the mean of the dependent variable, this suggests that the EOHO scheme can account for between 8% to 17% of all new infections during the period in which the scheme was active. This compares well with aggregate data from Public Health England that highlight that, during the weeks that the EOHO scheme was available, the share of infections traced to restaurants and food outlets increased from 5% to nearly 20%, subsequently declining again sharply with the end of the scheme (see Online Appendix Figure A2). Online Appendix Table A4 highlights that the results are robust to alternative functional forms.^6^

**Fig. 2.**
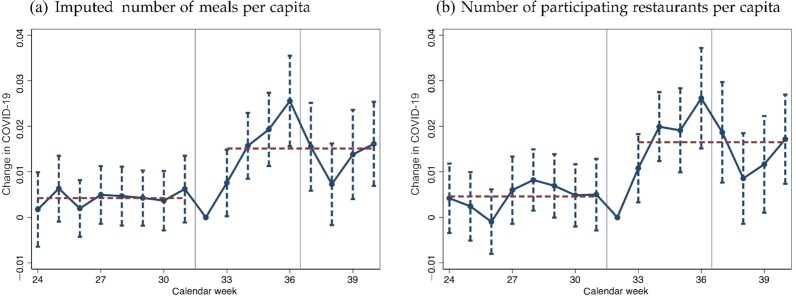
Difference-in-Differences and Parallel Trends Assumption: Impact of the EOHO Scheme on New COVID-19 Clusters. *Notes:* This figure presents regression estimates that capture the impact of EOHO exposure of an MSOA on the probability of a new COVID-19 cluster being detected over time. The regressions control for MSOA fixed effects and local-authority-district-by-week fixed effects. SEs are clustered at the district level with 90% confidence intervals shown. The dependent variable is a dummy that is equal to 1 in the case in which a new COVID-19 infection cluster was detected. A cluster is defined as at least two newly detected infections. Week refers to the week in which the specimen for the COVID-19 test was taken. New infection clusters increase sharply within a week of the introduction of the EOHO scheme and decline once again with the end of the scheme in MSOAs with more exposure to the scheme.

**Impact over time**: Figure 2 presents the impact of the EOHO scheme on infections over time. The results suggest that infection incidence started increasing among individuals taking a COVID-19 test one week after the scheme started in areas that had higher uptake, becoming statistically significant for tests taken in the second week, peaking in the last week the scheme was available, and, from then on, declined again. There is no evidence of any diverging pre-trends.^7^ The patterns map closely to observations from the aggregate restaurant bookings data given in Figure 1 as well as the patterns observed in the mobility data at the district level given in Online Appendix Figure A4.

**Robustness**: Online Appendix Table A5 highlights that results are robust to controlling for the potential time-varying impact of a large vector of area characteristics that have been suggested to be potential drivers of infections at the time. Online Appendix Table A6 shows that the results are robust to controlling for non-linear time trends across different subnational geographies: all nine NUTS1, 30 NUTS2 and 93 NUTS3 regions in columns (1)–(3). Columns (4)–(6) show that results are robust to controlling for non-linear time trends across politically relevant geographies at which level time-varying policies may be formulated: the 317 local authority districts and the 533 parliamentary constituencies. Online Appendix Figure A7 shows that results are not driven by any particular region performing a leave-on-one validation exercise, dropping all data pertaining to MSOAs that are part of England's nine, 30 and 93 NUTS1–NUTS3 regions.

Online Appendix A presents further analysis that documents very similar patterns in the take-up of the EOHO scheme as measured through anonymised individual-level transaction data from a large sample of UK issued payment cards that was made available to support research by the UK fintech Fable Data. The results, exploiting within-individual variation, document a notable increase in transactions in restaurants on EOHO days during weeks that the subsidy was available, with no other notable changes in consumer activity that may be indirectly induced by the scheme, with the exception—and not surprisingly—of notably reduced grocery store visits.

### 3.2. Exploiting Time Variation in Restaurant Visits

While the difference-in-differences results are very consistent with both the EOHO scheme’s design and timing, aggregate restaurant visit data and observed mobility measures, there remain concerns about whether we can interpret the results causally. To allay these concerns, I leverage data that measure inclement weather around the typical lunch and dinner hours during which people most likely frequent restaurants. The summer month of August, during which the EOHO discount was available, saw 59% more rain compared to the long-term average (Environment Agency, 2020).

I construct a measure that captures whether an area experienced notable rainfall during the prime lunch and dinner hours across different days over the time window the subsidy was available. This allows me to further exploit intra-day variation in the amount of rainfall that falls outside of regular hours during which one would visit restaurants.

#### 3.2.1. Rainfall on EOHO days and subsequent infections

Table 2 presents the main reduced-form estimates that link intra-day and inter-day rainfall measures to subsequent COVID-19 infections. Throughout this exercise, I estimate versions of specification (2) with different sets of rainfall measures. For ease of interpretation of the estimates, I discretise the rainfall measure to capture areas and time windows during which rainfall was in the upper decile.^8^

**Table 2.** Reduced-Form Impact of EOHO-Day Rainfall on the Emergence of Local COVID-19 Infection Clusters Later in the Week.

DV: any new COVID-19 cluster	(1)	(2)	(3)
*Panel A: data window covering exactly the EOHO scheme*			
Significant rainfall on EOHO days during lunch and dinner hours	−0.027$^{***}$	−0.027$^{***}$	−0.027$^{***}$
	(0.009)	(0.009)	(0.009)
Significant rainfall on non-EOHO days during lunch and dinner hours		0.001	
		(0.010)	
Significant rainfall on EOHO days outside lunch and dinner hours			−0.008
			(0.013)
Mean DV	0.143	0.143	0.143
Observations	33,955	33,955	33,955
Clusters	317	317	317
*Panel B: data window four weeks prior to the EOHO scheme (placebo)*			
Significant rainfall on EOHO days during lunch and dinner hours	−0.012	−0.012	−0.011
	(0.012)	(0.012)	(0.012)
Significant rainfall on non-EOHO days during lunch and dinner hours		−0.025$^{**}$	
		(0.011)	
Significant rainfall on EOHO days outside lunch and dinner hours			0.014
			(0.013)
Mean DV	0.058	0.058	0.058
Observations	27,164	27,164	27,164
Clusters	317	317	317
*Panel C: data window four weeks after the EOHO scheme (placebo)*			
Significant rainfall on EOHO days during lunch and dinner hours	−0.003	−0.004	−0.003
	(0.020)	(0.020)	(0.021)
Significant rainfall on non-EOHO days during lunch and dinner hours		0.016	
		(0.020)	
Significant rainfall on EOHO days outside lunch and dinner hours			0.020
			(0.023)
Mean DV	0.493	0.493	0.493
Observations	27,164	27,164	27,164
Clusters	317	317	317

*Notes:* This table presents difference-in-differences regression estimates of the impact of the EOHO scheme at the MSOA level on the emergence of new COVID-19 infection clusters during calendar weeks 32 to 36. All regressions also control for local-authority-by-week fixed effects. SEs are clustered at the district level with $^{***} p\lt 0.01$, $^{**} p\lt 0.05$, $^{*} p\lt 0.1$.

Panel A studies the impact of rainfall on subsequent COVID-19 infection clusters emerging. Column (1) suggests that an area that saw notable rainfall during the lunch and dinner hours on days during which the EOHO discount was available experienced, on average, 0.029 percentage points less new COVID-19 infection clusters—an effect of around 20% relative to the mean of the dependent variable. Column (2) shows that the effects are driven by lunch and dinner time rainfall measured during days on which the discount was available (but not for rainfall falling on other weekdays). Lastly, column (3) documents the rainfall that falls *on the same weekdays* during which the EOHO discount was offered—but outside the lunch and dinner hours—showing that rainfall falling outside the lunch and dinner hours has no effect on infections.

In panels B and C I perform the same exercises, but focus on data pertaining to the four-week windows before and after the EOHO scheme was available, finding no such consistent pattern as documented in panel A.

It is worth highlighting that this suggests that the specific design of the EOHO scheme, by concentrating restaurant visits on a few days within a week, may be particularly relevant in helping understand how it facilitated the spread of COVID-19.

In Online Appendix Table A7 I show that there are no lead effects and a weak lagged effect. In Online Appendix Table A8 I present alternative rainfall measures that specifically measure rainfall simply in overall levels—the results are very similar. Lastly, Online Appendix Table A9 shows that the results are robust to using alternative measures of COVID-19 infections.

#### 3.2.2. Inclement weather and mobility patterns

**Fig. 3.**
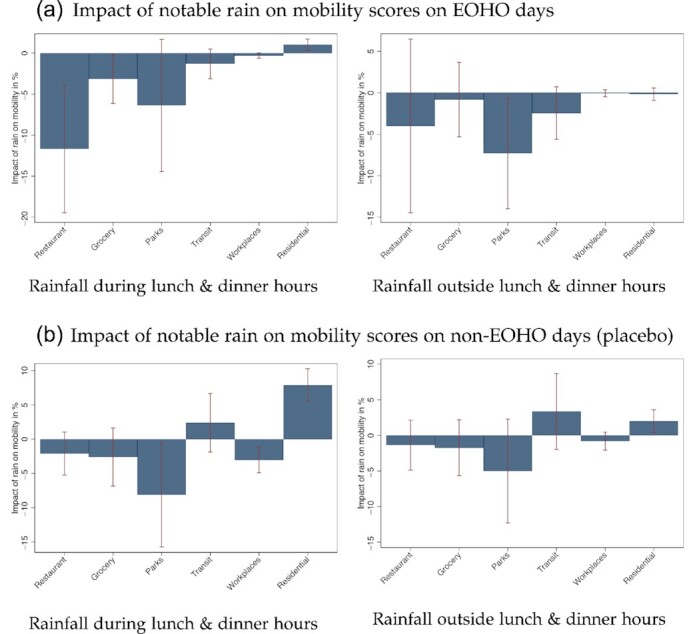
Impact of Intra-Day Rainfall on Daily Mobility in Weeks 32 to 36 on EOHO Days and on Non-EOHO Days. *Notes:* This figure visualises the results of the regressions presented in Online Appendix Table A10. The bar charts represent the estimated impact of notable rainfall during lunch and dinner hours (left column) or rainfall falling outside lunch and dinner hours (right column) on Google mobility during calendar weeks 32 to 36 when the EOHO program ran. The effects are expressed in percentages relative to the mean of the mobility score. Panel (a) presents estimates for these impacts on days during which the EOHO discount was available (Mondays–Wednesdays), while panel (b) focuses on the weekdays during which the discount was not available (Thursdays–Sundays). The regressions control for district fixed effects and NUTS2-region-by-date fixed effects. The 90% confidence intervals obtained from clustering SEs at the district level are indicated.

Lastly, using daily district-level mobility data from Google (2020), I show that inclement weather does affect people's movements—in particular, restaurant visits—in patterns that are very consistent with the results on infections. The mobility data, while clustering specific recreation activities together, have the advantage of being temporarily more granular daily data, but are spatially coarser. The estimated effects of rainfall on mobility are presented visually in Figure 3 (point estimates are available in Online Appendix Table A10).

Figure 3(a) presents the empirical relationship between notable rainfall—either during lunch and dinner hours (left column) or outside lunch and dinner hours (right column)—on the Mondays to Wednesdays during calendar weeks 32 to 36 when the EOHO discount was available on Google mobility scores by mobility type. The results suggest that lunch and dinner time rainfall reduces the mobility score capturing restaurant visits by up to 20%. Similarly, we see notable declines of around 10% for time spent in parks. We observe marginal increases in time spent at home, but null effects on mobility measures that proxy for time spent shopping, in transit or at workplaces. The results in the right column suggest that rainfall outside lunch and dinner hours on the same days does not appear to have an effect on any of the mobility measures.

Panel B presents the placebo estimates that focus on the weekdays during which the discount was not available (Thursdays–Sundays). While the notable rainfall during the lunch and dinner hours has a similar effect on time spent in parks, it has a much weaker and just statistically insignificant negative effect on the mobility measure capturing restaurant visits. This is further suggestive evidence highlighting that the EOHO scheme drastically increased restaurant visits on Mondays–Wednesdays, but less so in places that experienced adverse weather.

Online Appendix Table A11 focuses exclusively on the Google mobility measure picking up restaurant visits, but also adds the placebo exercises studying the four-week windows before and after the EOHO scheme ran, mimicking the analysis of infections presented in Table 2. The results are very consistent. The absence of an empirical link between rainfall and restaurant visits on EOHO days during the four-week periods before and after the scheme ran is further corroborating evidence suggesting that the EOHO scheme, by shifting and drastically increasing restaurant visits (as opposed to affecting mobility more broadly), is responsible for a marked uptick in infections.

### 3.3. Quantification in Absolute Numbers

The above estimates have been presented in relative numbers. We can also provide a quantification in absolute numbers of cases, taking the empirical estimates from the various exercises as the basis. This is provided in Online Appendix Table A12. Throughout the different empirical specifications with different fixed effects, different functional forms or different measures of the dependent variables are presented in Table 1 and Online Appendix Table A4. The estimates are remarkably stable and similar, indicating that the EOHO scheme has caused between 10.5%–14.6% of all infections that were detected between calendar weeks 32 to 36 inclusive. These ranges capture the lowest value implied with 95% confidence intervals of the empirical estimates and the highest 95% confidence interval of the estimates across specifications.^9^ Nevertheless, there are good reasons to believe that this estimate, which is based on confirmed infections, understates the true impact. There are strong indications that uptake of the EOHO scheme is estimated to have been 2.5 times higher in the 18-to-34 demographic vis-à-vis the over-55s cohort. Given the fact that there is a notably higher likelihood of asymptomatic or subclinical COVID-19 (see, e.g., Davies *et al*., 2020) among the younger demographic, this may result in many infections not being detected, implying that the estimate could constitute a lower bound.^10^ To arrive at an estimate of the number of cases in absolute terms, we require an estimate of the overall number of cases. There are at least two measures of infections during calendar weeks 32 to 36. First, England saw a total of 45,382 lab-confirmed infections with a test date between calendar weeks 32 and 36. A second estimate is provided by the Office of National Statistics that has carried out a testing program in representative samples of the population. This suggests that calendar weeks 32 to 36 inclusive saw a total number of infections in the English population ranging, with 95% confidence, between 73,400 and 149,100 infections.^11^ The figures suggest that the EOHO scheme may have caused between between 4,798 and 6,643 symptomatic infections or 7,759 and 21,824 overall infections, including asymptomatic cases directly. This is captured in panel C of Online Appendix Table A12.

This estimate is unlikely to capture the full pandemic impact of the EOHO scheme as this will spread well beyond calendar weeks 32 to 36. First, the estimates consider only the period from calendar week 32 to calendar week 36, while Figure 2 suggests that there was still a declining but economically and statistically significant impact of the EOHO scheme even in week 37 and in week 38. Second, the above estimates are unlikely to capture the full chain of onward infections unless the geographic distribution of such onward infections matches exactly that of the index cases.

To illustrate the relevance of onward infections on the absolute numbers, even if just for illustrative purposes, panel D presents some computations that leverage the above point estimates, assuming 0.5 onward infections per case over a four-week window following the end of the EOHO scheme.^12^ It highlights that the dynamic impact of the EOHO scheme may be more significant than is implied by the static point estimates. The above conservative assumptions would indicate that the EOHO scheme may have caused up to 69,008 infections directly and indirectly between calendar weeks 32 and 40. Naturally, this is not an epidemiological model, but serves to illustrate the point that infection numbers may appear low at first sight due to the dynamic nature of the pandemic and the indirect effects of onward infection chains.

## 4. Conclusion

Policy makers are debating the optimal policy response to the COVID-19 pandemic. The economic impact of changed consumer behaviour in response to rising and falling COVID-19 infections is far from uniformly distributed across sectors (Barrot *et al*., 2020; Brinca *et al*., 2020; Carvalho *et al*., 2020; Dingel and Neiman, 2020). The UK’s policy response in the wake of the first wave of the COVID-19 pandemic shared many features of other countries' fiscal response. The most prominent point of divergence between the UK’s fiscal response and that of other countries was a large-scale demand-inducing measure targeted at the restaurant sector. A total of GBP 850 million was spent to subsidise the cost of eating out by up to 50% in the month of August. This came at a time when epidemiological studies suggested that restaurant dining may be a particularly risky setting. This paper shows that the eat-out-to-help-out scheme, hailed as a boon for the ailing sector, causally increased COVID-19 community transmission. By subsidising an economic activity associated with negative health externalities, the estimates suggest that the eat-out-to-help-out scheme may have been responsible for between 8%–17% of all newly detected COVID-19 infections (and likely many more non-detected asymptomatic infections) in late summer. This highlights the fact that fiscal responses aimed to cushion the economic fallout from COVID-19 have to pay particular attention to epidemiological risks as, otherwise, they may significantly worsen the pandemic progression and undermine any short-term economic benefits.

Additional Supporting Information may be found in the online version of this article:

**Online Appendix**

**Replication Package**

## Supplementary Material

ueab074_Online_Appendix The data and codes for this paper are available on the Journal repository. They were checked for their ability to reproduce the results presented in the paper. The authors were granted an exemption to publish their data because access to the data is restricted. However, the authors provided the Journal with temporary access to the data, which allowed the Journal to run their codes. The codes are available on the Journal repository. The data and codes were checked for their ability to reproduce the results presented in the paper. The replication package for this paper is available at the following address: https://doi.org/10.5281/zenodo.5257664.

## Footnotes

1 MSOAs are statistical geographies that are constructed to produce spatial units with comparable population and household numbers, making them particularly suitable for econometric exercises and reducing the need for population normalisations or weighting of data.

2 COVID cases are tagged to the MSOA of residence. The implicit assumption made here is that consumers are more likely to take up the EOHO scheme in restaurants nearby, rendering local take-up measures adequate to measure the impact on infections. The restaurant finder data can be accessed at https://github.com/hmrc/eat-out-to-help-out-establishments.

3 Participating chains are listed on https://web.archive.org/web/20200831011834/https://www.tax.service.gov.uk/eat-out-to-help-out/find-a-restaurant/restaurant-chains.

4
Online Appendix Table A1 shows that, while there are other changes in mobility, these are relatively marginal. For the empirical exercise at the more granular MSOA level, we fully account for mobility and policy changes by flexibly controlling for district-by-week fixed effects.

5 Naturally, even if the overall number of restaurant visits may have remained the same across the week, the concentration and shifting of visits within the week may have significantly increased the epidemiological risk associated with restaurant visits.

6 One alternative explanation could be that there is differential testing intensity that endogenously responds to EOHO take-up both due to supply and demand side factors. In aggregate, the number of tests carried out over the period was quite constant at around 150,000 PCR tests per day. Furthermore, while it may be that areas with higher EOHO take-up have more test capacity and could explain this observation, it is unlikely to consistently do so for the second analysis that explores the intensive margin of EOHO uptake.

7
Online Appendix Figure A6 shows that results are broadly carried across reasonably different post-treatment time windows.

8
Online Appendix Table A8 presents the same table for alternative rainfall measures, yielding very similar results.

9 This figure squares extremely well with PHE data of new infection clusters that were detected and traced to an origin (which is the vast minority of all infections), presented in Online Appendix Figure A2, which increased by a similar amount.

10 Polling data suggest that the EOHO scheme was predominantly used by the 18-to-34 demographic, who made up 41% of customers, while just 17% of over-55s took advantage of the offer; see https://bit.ly/3dYijmr.

11 The advantage of the representative sample-based estimate is that it also captures asymptomatic infections. The ratio of detected to non-detected cases ranges between 1.61 and 3.28, which is not incredible given population estimates that between 20% to 70% of individuals may be infectious but asymptomatic (see Yanes-Lane *et al*., 2020), who, naturally, could cause onward infections.

12 Since the reproduction rate estimates around the time were increasing from 0.9 in early August to 1.5 by the end of September, suggesting that each case causes between 0.9 to 1.5 additional infections, this appears conservative.

## Notes

I would like to thank Arun Advani, Mirko Draca, Claudia Eger, Jakob Fetzer, Thomas Graeber, Simon Martin, Christopher Roth, Andreas Stegmann, Pedro CL Souza for comments and suggestions on this draft. This work also benefited from the author being part of the Royal Society’s DELVE Initiative.
