# Supplementary material for: Subsidising the spread of COVID-19: Evidence from the UK’S Eat-Out-to-Help-Out Scheme*
Source: Econ J (London). 2021 Oct 26:ueab074. doi: 10.1093/ej/ueab074 (PMC8574521; doi:10.1093/ej/ueab074)
Supplement: ueab074_Online_Appendix — The data and codes for this paper are available on the Journal repository. They were checked for their ability to reproduce the results presented in the paper. The authors were granted an exemption to publish their data because access to the data is restricted. However, the authors provided the Journal with temporary access to the data, which allowed the Journal to run their codes. The codes are available on the Journal repository. The data and codes were checked for their ability to reproduce the results presented in the paper. The replication package for this paper is available at the following address: https://doi.org/10.5281/zenodo.5257664. [file ueab074_online_appendix.pdf]

# Appendix to "Subsidizing the spread of COVID19: Evidence from the UK"

For Online Publication June 9, 2021

Thiemo Fetzer

## A Take-up evidence from anonymized individual-level transaction data

To corroborate the evidence of take-up of EOHO this paper leverages additional data capturing consumer demand more closely. Specifically, individual level anonymised transaction data from the UK Fintech Fable Data is leveraged in this appendix to document how EOHO increased demand for restaurant visits around the days and during weeks in which the subsidy was available.

This will directly corroborate the evidence from the Google Mobility data analysis and also shed some light suggesting that the results are unlikely to be confounded by other changes in mobility. Further, it corroborates the work of **?** suggesting both little persistence and no notable spillover effects on other types of consumer spending – with the exception of reduced grocery shopping. As EOHO was anecdotally motivated by the wish to encourage wider economic activity, such as visiting of other retail outlets, this is suggestive that it may have failed to do so in delivering on this expectation.

**Data and analysis** The anonymized individual level transaction level data is collapsed to an unbalanced individual-level daily panel data set measuring the number of transaction across different types of vendors. Fable has classified the vendors into broad categories with restaurant and hospitality venues being classed as "Food and Beverage", clothing retailers being classified as "Clothing & Apparel" and general online marketplaces being classified as "General Merchandise", while grocery stores and chains would be labelled as such. The temporal granularity along with the granularity of the transaction types enables us to document more sharply the impacts that the scheme had on consumer behavior in a quite demanding empirical design.

Specifically, we estimate the following empirical specification:

$$y_{i,t} = \nu_i + \gamma_{l(i),w(t)} + \text{EOHO}_{d(t)} + \eta \times Post_t \times \text{EOHO}_{d(t)} + \epsilon_{d,t} \quad (1)$$

Here, $y_{i,t}$ measures number of transactions on a date $t$ in a specific spending category. The regression exploits *within individual variation* by absorbing individual

level fixed effects $\nu_i$ along with controlling for local authority by week fixed effects $\gamma_{l(i),t}$. The coefficient of interest is $\eta$, which captures the differential changes in $y_{i,t}$ on Monday-Wednesday during which the scheme was available during calendar weeks 32 to 36.

As with the main estimation we can pool the effect across weeks 32 to 36 to present results in tabular format, we can, however, also present results in visual format to provide additional evidence in support of the common trends assumption.

**Results** Appendix Table A2 documents that individual card use on days during which the subsidy was available saw a notable increase in transactions in Food and Beverage outlets – with an increase of activity by 8.8% relative to the baseline mean. There are no noticeable other effects on consumer activity with the exception of there being a significant decline in transactions for groceries, suggesting that EOHO-induced restaurant visits were substituting – not surprisingly – for spending on groceries. This suggests that there have been no other general patterns suggesting changes in consumer demand that may have shifted the risk exposure profile.

To complement the mobility analysis I have also incorporated the event study in Appendix Figure A5. This figure highlights that individual card transactions in Food and Beverage outlets strongly increased over the EOHO period and then declined again with the program ending, with no discernible pre-trends, again following the main results in the paper.

# B Additional Figures and Tables

Figure A1: COVID19 spread across MSOA's in England

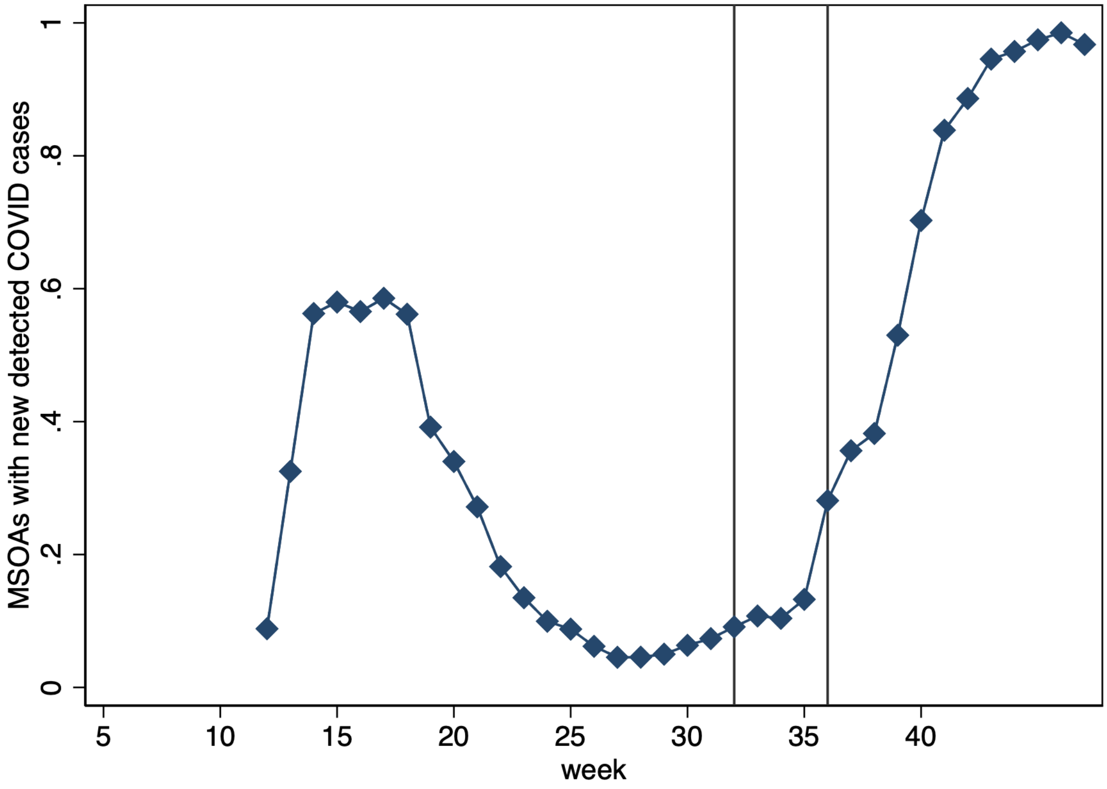


**Notes:** Figure plots the share of English MSOA's that report at least three new cases of COVID19 per calendar week. The vertical lines indicate the time that the Eat-Out-To-Help-Out scheme was open.

Figure A2: Share of COVID19 infections that have been contact traced to restaurants

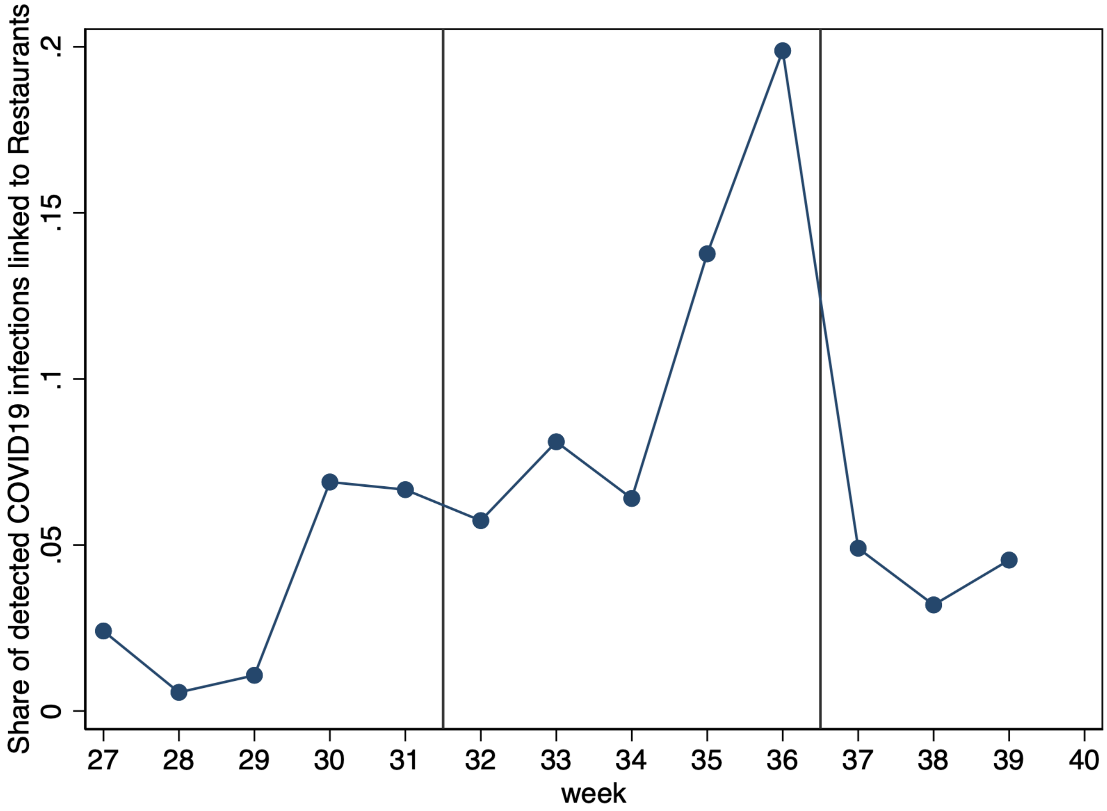


**Notes:** Figure plots data provided by the weekly COVID19 situation reports from Public Health England. During weeks 32 to 36, PHE identified 739 COVID19 infection incidents that were traced back to a specific origin. The figure plots the share of these incidents attributable to Food outlet/restaurants. The share drastically increases from around 5% to around 20% in week 36 and subsequently declining again after the EOHO program ended. The PHE data is very incomplete. During calendar weeks 32 to calendar week 36 more than 50,000 COVID19 cases were detected highlighting that PHE was able to identify only a small share of the infections.

Figure A3: Number of restaurant premises registered to participate in the Eat-Out-To-Help-Out scheme across England

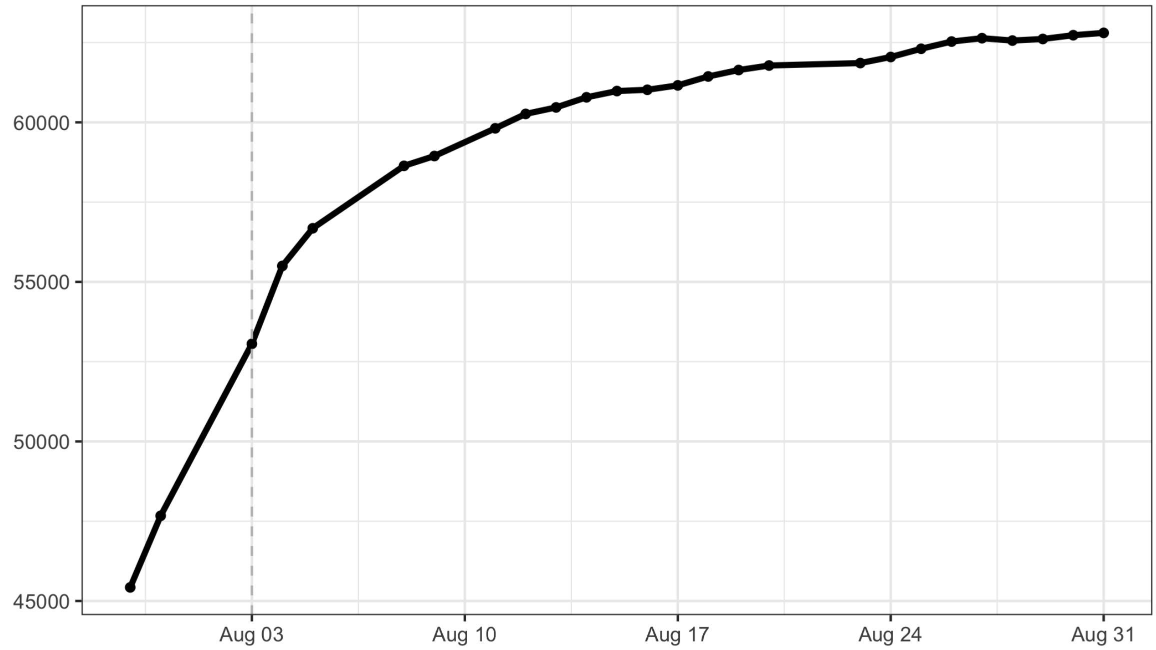


**Notes:** Time series plots the number of restaurants premises that are registered in the scheme at different points in time in England. The bulk of registrations is from HMRC's public github repository. Chain restaurant premises are added separately for completeness. Their inclusion does not affect the results substantially. The program started on Aug 3, 2020 and lasted until Aug 31, 2020. Dots indicate points where a flat file with the restaurants was downloadable from the HMRC Github repository track changes. The data in between is interpolated.

Figure A4: Differential increase in (likely) Restaurant visits on Monday-Wednesday during EOHO as measured by Google Mobility data

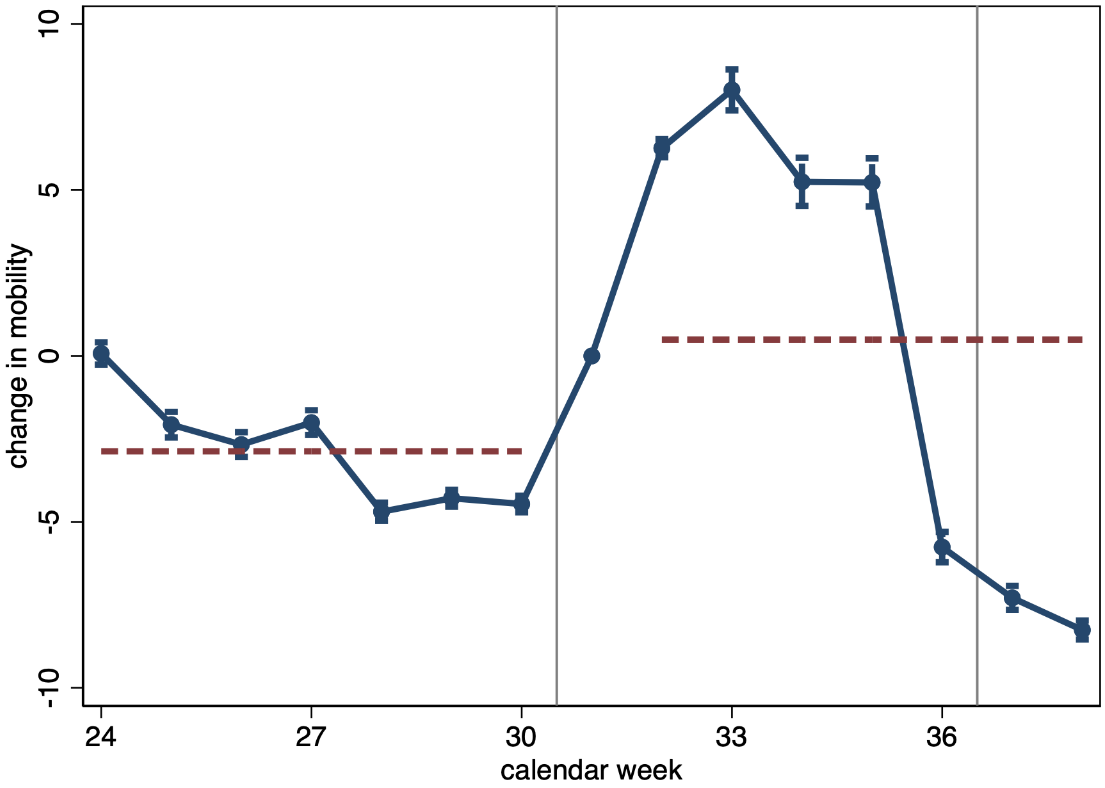


**Notes:** Figure plots the change in Google mobility measure on days during which the EOHO scheme was active (Monday, Tuesday, Wednesday) before and after the scheme was introduced from calendar week 32 inclusive onwards up until Monday 31, August inclusive in calendar week 36. The regression controls for district fixed effects, district-specific linear trends by calendar week and day of week fixed effects. 90% standard errors obtained from clustering standard errors at the district level are indicated.

Figure A5: Event Study: Impact of EOHO on transaction for Food and Beverage outlets over time

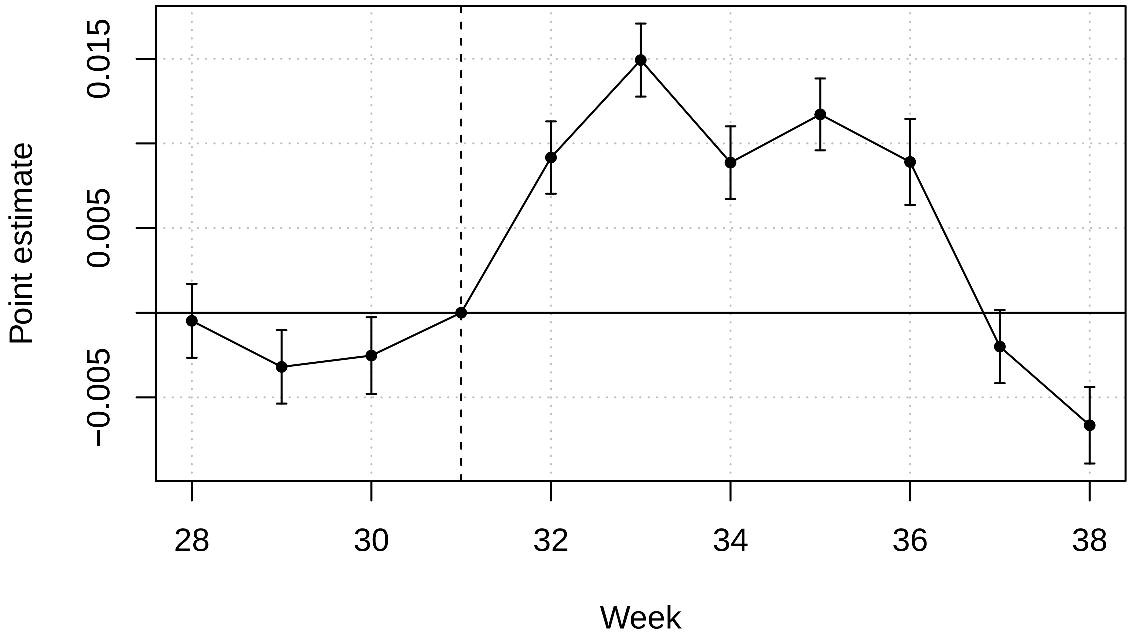


**Notes:** Figure plots regression results using individual anonymized card activity data from Fable Data. The point estimates represent the differential change in transactions for Food and Beverage outlets across calendar weeks from Mondays to Wednesdays when the EOHO discount was available from week 32 to week 36. All regressions include individual level fixed effects, district by week fixed effects and weekday fixed effects. 95% confidence intervals obtained from clustering standard errors two way at the date and district level are indicated.

Figure A6: Robustness to dropping post-treatment weeks

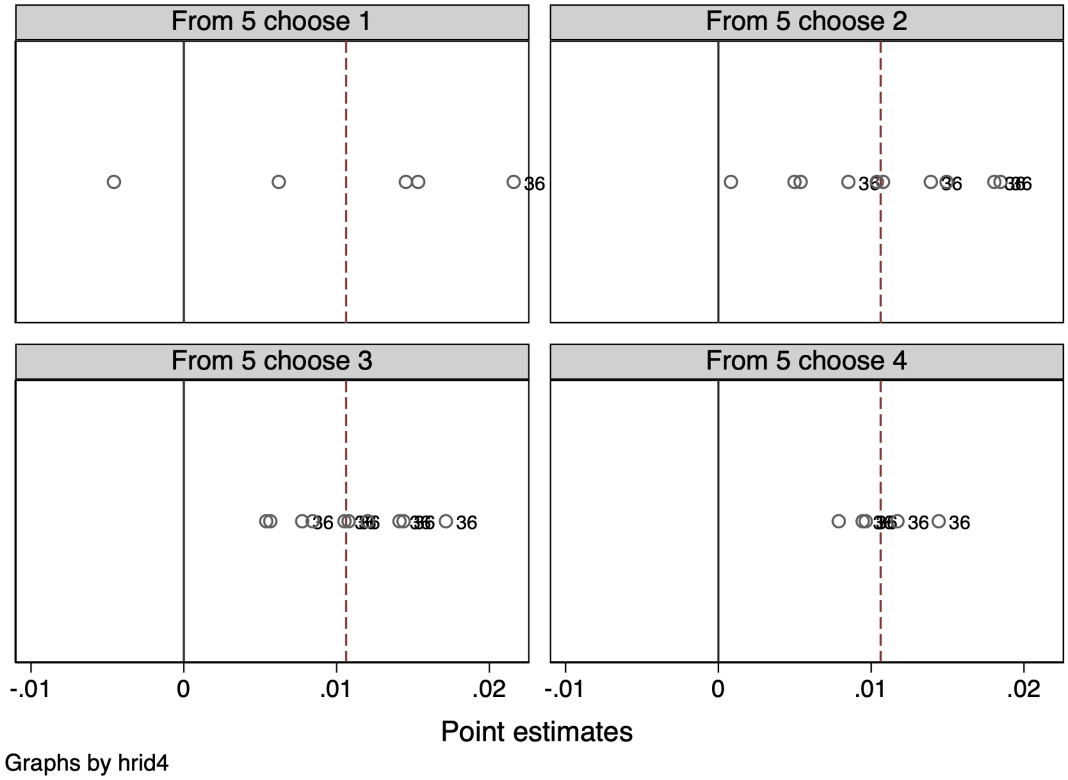


**Notes:** Figure plots the results of all permutations of dropping data pertaining to the post treatment weeks 32-36 from the analysis of specification (3) in Panel A of Table **??**). In total, there are five weeks post treatment. The different figure panels provide the distribution of all possible estimated coefficients that are obtained after dropping 1, 2, 3, or 4 weeks of post-treatment data. The solid black line indicates zero, while the dashed line indicates the point estimate that is estimated using all post-treatment weeks. The results are only sensitive if only calendar weeks 32 and 33 are considered by themselves. Given the epidemiological lags and slowly expanding take-up of EOHO this is not surprising. The point estimates are higher if the post-treatment window includes calendar week 36 but results are carried also without that specific week.

Figure A7: Distribution of point estimates obtained when dropping one region a time

*Panel A*: Dropping each of the 9 NUTS1 regions in turn

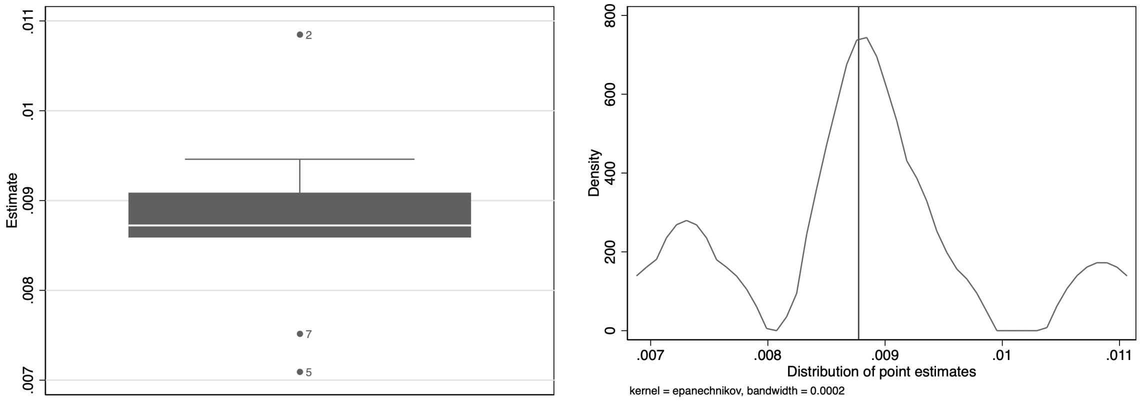


*Panel B*: Dropping each of the 30 NUTS2 regions in turn

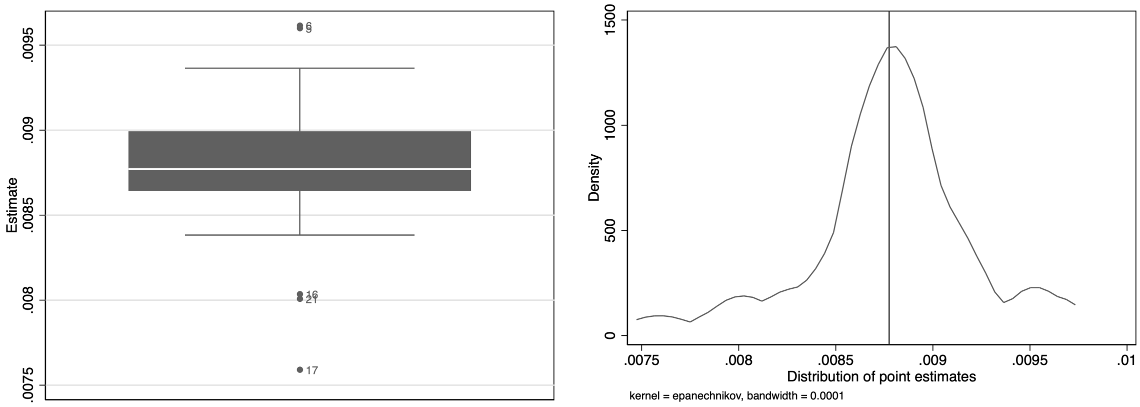


*Panel C*: Dropping each of the 93 NUTS3 regions in turn

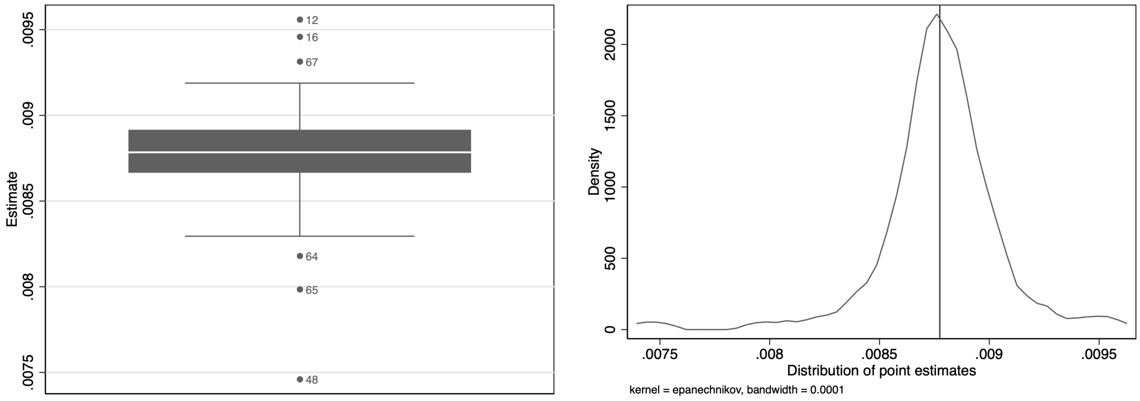


**Notes:** Figures present the distribution of the point estimates obtained when dropping one region a time. The estimating regression has as dependent variable an indicator that is equal to 1 in case a new COVID19 cluster of more than two cases was detected in an MSOA. The regressions include MSOA fixed effects and district by time fixed effects. The coefficient estimate is the interaction between the post indicator marking the start of the EOHO scheme and the log of the number of number of restaurants +1 divided by the MSOA population. Standard errors are clustered at the district level.

Table A1: EOHO and Google Mobility

| DV: Google mobility in | Retail & Recreation | Grocery | Parks | Transit | Workplace | Residential |
|---|---|---|---|---|---|---|
| | (1) | (2) | (3) | (4) | (5) | (6) |
| Post Week 32 × EOHO Weekday | 6.780*** | -0.615*** | 6.489*** | -1.295*** | -2.625*** | 0.059** |
| | (0.253) | (0.100) | (1.178) | (0.258) | (0.077) | (0.023) |
| Mean DV | -31.568 | -10.997 | 69.819 | -31.403 | -38.000 | 11.908 |
| Observations | 24061 | 24597 | 17023 | 24224 | 26925 | 26331 |
| Clusters | 312 | 311 | 300 | 311 | 312 | 311 |

**Notes:** Table presents difference-in-difference regression estimates studying the evoltution of Google mobility measures at the district level over time between calendar weeks 24 and 36. The EOHO scheme was active on Mondays, Tuesdays and Wednesdays from August 3, 2020 to August 31, 2020 (calendar weeks 32 to 36). The dependent variable is the mobility measure relative to pre COVID19 levels per day across the categories provided by Google indicated in the column head. The regressions control for district FE, week fixed effects, and weekday fixed effects. Standard errors are clustered at the district level with starts indicating *** p< 0.01, ** p< 0.05, * p< 0.1.

Table A2: Impact of EOHO on consumer spending by type

| | Food & Beverage | General Merchandize | Groceries | Health & Beauty | Leisure | Travel |
|---|---|---|---|---|---|---|
| EOHO day × EOHO week | 0.027*** | -0.006 | -0.012*** | -0.004 | 0.000 | -0.000 |
| | (0.005) | (0.007) | (0.005) | (0.003) | (0.001) | (0.001) |
| N | 7586231 | 7586231 | 7586231 | 7586231 | 7586231 | 7586231 |
| Card FE | X | X | X | X | X | X |
| Region x Week FE | X | X | X | X | X | X |
| Day of Week FE | X | X | X | X | X | X |
| Mean | 0.305 | 0.568 | 0.369 | 0.039 | 0.029 | 0.133 |

**Notes:** Table presents regression results showing the impact of EOHO on consumer spending as measured through anonymised individual-level card transaction data from Fable Data. All regressions control for card fixed effects, region by week fixed effects and day of week fixed effects. Standard errors, presented in parantheses, are clustered twoway by district and date with stars indicating * p < 0.1, ** p < 0.05, *** p < 0.01.

Table A3: Clustering of standard errors at different spatial levels: Impact of EOHO on Emergence of Local Infection Clusters

| DV: Any new COVID19 cluster | (1) | (2) | (3) | (4) | (5) | (6) |
|---|---|---|---|---|---|---|
| ***Panel A:*** Clustering at the local authority district level | | | | | | |
| Post $\times$ log(EOHO covered meals per capita) | 0.007*** | 0.007*** | 0.009*** | 0.009*** | 0.007*** | 0.007*** |
| | (0.003) | (0.003) | (0.003) | (0.003) | (0.002) | (0.002) |
| Mean DV | 0.096 | 0.096 | 0.096 | 0.096 | 0.096 | 0.096 |
| Observations | 88283 | 88283 | 88283 | 88283 | 88283 | 88283 |
| MSOA | 6791 | 6791 | 6791 | 6791 | 6791 | 6791 |
| Clusters | 317 | 317 | 317 | 317 | 317 | 317 |
| ***Panel B:*** Clustering at at NUTS3 region level | | | | | | |
| Post $\times$ log(EOHO covered meals per capita) | 0.007** | 0.007*** | 0.009*** | 0.009*** | 0.007*** | 0.007*** |
| | (0.003) | (0.003) | (0.003) | (0.003) | (0.003) | (0.003) |
| Mean DV | 0.096 | 0.096 | 0.096 | 0.096 | 0.096 | 0.096 |
| Observations | 88283 | 88283 | 88283 | 88283 | 88283 | 88283 |
| MSOA | 6791 | 6791 | 6791 | 6791 | 6791 | 6791 |
| Clusters | 93 | 93 | 93 | 93 | 93 | 93 |
| ***Panel C:*** Clustering at at NUTS2 region level | | | | | | |
| Post $\times$ log(EOHO covered meals per capita) | 0.007** | 0.007*** | 0.009*** | 0.009*** | 0.007*** | 0.007*** |
| | (0.003) | (0.003) | (0.002) | (0.003) | (0.002) | (0.002) |
| Mean DV | 0.096 | 0.096 | 0.096 | 0.096 | 0.096 | 0.096 |
| Observations | 88283 | 88283 | 88283 | 88283 | 88283 | 88283 |
| MSOA | 6791 | 6791 | 6791 | 6791 | 6791 | 6791 |
| Clusters | 30 | 30 | 30 | 30 | 30 | 30 |
| **Area by Week FE**: | NUTS2 | NUTS3 | LAD | NUTS2 | NUTS3 | LAD |

**Notes:** Table presents difference-in-difference regression estimates studying the impact of the EOHO at the MSOA level on the emergence of new COVID19 infection clusters across the 13 calendar weeks from 24 to 36. A COVID19 infection cluster is defined as a week in which there were strictly more than two new COVID19 infections detected in tests taken during the week. All regressions control for MSOA-level fixed effects. The regression also control for time fixed effects specific to each NUTS2, NUTS3 or local authority specific week fixed effects. Standard errors are clustered at the level indicated in the panel head with starts indicating *** p< 0.01, ** p< 0.05, * p< 0.1.

Table A4: Robustness of Impact of EOHO on Emergence of Local Infection Clusters: Alternative functional forms

| DV: indicated in panel label | (1) | (2) | (3) | (4) | (5) | (6) |
|---|---|---|---|---|---|---|
| ***Panel A:*** Dependent variable: Any COVID19 cluster | | | | | | |
| Post × log(EOHO covered meals per capita) | 0.007*** | 0.009*** | 0.009*** | | | |
| | (0.003) | (0.003) | (0.003) | | | |
| Post × log(EOHO restaurants per capita) | | | | 0.008*** | 0.009*** | 0.011*** |
| | | | | (0.003) | (0.003) | (0.003) |
| Mean DV | 0.096 | 0.096 | 0.096 | 0.096 | 0.096 | 0.096 |
| Observations | 88283 | 88283 | 88283 | 88283 | 88283 | 88283 |
| MSOA | 6791 | 6791 | 6791 | 6791 | 6791 | 6791 |
| Additional controls | 390 | 1209 | 4121 | 390 | 1209 | 4121 |
| Clusters | 317 | 317 | 317 | 317 | 317 | 317 |
| ***Panel B:*** Dependent variable: log(# of COVID19 cases in cluster) | | | | | | |
| Post × log(EOHO covered meals per capita) | 0.012** | 0.015*** | 0.015*** | | | |
| | (0.005) | (0.005) | (0.005) | | | |
| Post × log(EOHO restaurants per capita) | | | | 0.011** | 0.013*** | 0.016*** |
| | | | | (0.005) | (0.005) | (0.005) |
| Mean DV | 0.167 | 0.167 | 0.167 | 0.167 | 0.167 | 0.167 |
| Observations | 88283 | 88283 | 88283 | 88283 | 88283 | 88283 |
| MSOA | 6791 | 6791 | 6791 | 6791 | 6791 | 6791 |
| Additional controls | 390 | 1209 | 4121 | 390 | 1209 | 4121 |
| Clusters | 317 | 317 | 317 | 317 | 317 | 317 |
| ***Panel C:*** Dependent variable: Inverse hyperbolic sine (asinh) of # of COVID19 cases in cluster | | | | | | |
| Post × log(EOHO covered meals per capita) | 0.015** | 0.019*** | 0.020*** | | | |
| | (0.006) | (0.006) | (0.007) | | | |
| Post × log(EOHO restaurants per capita) | | | | 0.014** | 0.018*** | 0.021*** |
| | | | | (0.006) | (0.006) | (0.006) |
| Mean DV | 0.214 | 0.214 | 0.214 | 0.214 | 0.214 | 0.214 |
| Observations | 88283 | 88283 | 88283 | 88283 | 88283 | 88283 |
| MSOA | 6791 | 6791 | 6791 | 6791 | 6791 | 6791 |
| Additional controls | 390 | 1209 | 4121 | 390 | 1209 | 4121 |
| Clusters | 317 | 317 | 317 | 317 | 317 | 317 |
| **Area by Week FE**: | NUTS2 | NUTS3 | LAD | NUTS2 | NUTS3 | LAD |

**Notes:** Table presents difference-in-difference regression estimates studying the impact of the EOHO at the MSOA level on the emergence of new COVID19 infection clusters across the 13 calendar weeks from 24 to 36. The dependent variable is 1 in case an MSOA reported more than two new detected infections per calendar week. The independent variable in panel A measures the EOHO scheme as the log number of meals served in restaurants in an MSOA that participate in the EOHO scheme plus 1 divided by the population in the area. The independent variable in panel B measures the EOHO scheme as the log number of restaurants that participate in the EOHO scheme in an MSOA plus 1 divided by the population in the area. The specifications across panels explore the robustness to controlling for more granular non-linear time fixed effects. NUTS refers to the nomenclature unitÃ©s territoriales statistiques which subdivides the England into 11, 30 and 93 regions. LAD refers too local authority districts. PCON refers to Westminster parliamentary constituencies. Standard errors are clustered at the district level with starts indicating *** p< 0.01, ** p< 0.05, * p< 0.1.

Table A5: Robustness of Impact of EOHO on Emergence of Local Infection Clusters: Additional non-parametric control variables

| DV: Any new COVID19 cluster | (1) | (2) | (3) | (4) | (5) | (6) |
|---|---|---|---|---|---|---|
| ***Panel A:*** Measuring EOHO by imputed meals per capita | | | | | | |
| Post × log(EOHO covered meals per capita) | 0.009*** | 0.009*** | 0.008*** | 0.007** | 0.006** | 0.005** |
| | (0.003) | (0.003) | (0.003) | (0.003) | (0.003) | (0.003) |
| Mean DV | 0.096 | 0.096 | 0.096 | 0.096 | 0.096 | 0.096 |
| Observations | 88283 | 88270 | 88270 | 88270 | 88270 | 88270 |
| Clusters | 317 | 316 | 316 | 316 | 316 | 316 |
| ***Panel B:*** Measuring EOHO by number of restaurants per capita | | | | | | |
| Post × log(EOHO restaurants per capita) | 0.011*** | 0.010*** | 0.010*** | 0.008*** | 0.006** | 0.006** |
| | (0.003) | (0.003) | (0.003) | (0.003) | (0.003) | (0.003) |
| Mean DV | 0.096 | 0.096 | 0.096 | 0.096 | 0.096 | 0.096 |
| Observations | 88283 | 88270 | 88270 | 88270 | 88270 | 88270 |
| Clusters | 317 | 316 | 316 | 316 | 316 | 316 |
| **Week x Additional control**: | | | | | | |
| Population density measures | | X | X | X | X | X |
| Spring 2020 COVID19 exposure | | | X | X | X | X |
| Commuting exposure | | | | X | X | X |
| Student exposure | | | | | X | X |
| Tenure types | | | | | | X |

**Notes:** Table presents difference-in-difference regression estimates studying the impact of the EOHO at the MSOA level on the emergence of new COVID19 infection clusters across the 13 calendar weeks from 24 to 36. The dependent variable is 1 in case an MSOA reported more than two new detected infections per calendar week. The specifications across panels explore the robustness to adding additional successively more MSOA-level control variables interacted with week fixed effects to account for non-linear trends in these measures. Population density measures include: population density, the standard deviation of population density across lower-level super output areas (LSOAs) that make up the MSOA, and the area size of the MSOA in km2. Spring 2020 COVID measures an MSOA's exposure to COVID from March to July 2020 as the number of COVID19 deaths per capita ; the number of COVID19 cases per capita; the number of non-COVID19 deaths per capita and the share of COVID19 deaths among all deaths. Commuting exposure measures based on 2011 census the number of people usually commuting for work into an MSOA divided by the MOSA's population; the number of commuters usually resident but commuting elsewhere divided by the MSOA's population. Student exposure measures based on the 2011 census the share of full time students resident in an MSOA. Tenure types measures the share of households living in rented or owned accommodation. All regressions also control for local authority by week fixed effects. Standard errors are clustered at the district level with starts indicating *** $p < 0.01$, ** $p < 0.05$, * $p < 0.1$.

Table A6: Robustness of Impact of EOHO on Emergence of Local Infection Clusters: Alternative fixed effects

| DV: Any new COVID19 cluster | (1) | (2) | (3) | (4) | (5) | (6) |
|---|---|---|---|---|---|---|
| ***Panel A:*** Measuring EOHO by imputed meals per capita | | | | | | |
| Post $\times$ log(EOHO covered meals per capita) | 0.007*** | 0.007*** | 0.009*** | 0.009*** | 0.007*** | 0.007*** |
| | (0.003) | (0.003) | (0.003) | (0.003) | (0.002) | (0.002) |
| Mean DV | 0.096 | 0.096 | 0.096 | 0.096 | 0.096 | 0.096 |
| Observations | 88283 | 88283 | 88283 | 88283 | 88283 | 88283 |
| MSOA | 6791 | 6791 | 6791 | 6791 | 6791 | 6791 |
| Additional controls | 117 | 390 | 1209 | 4121 | 6929 | 9373 |
| Clusters | 317 | 317 | 317 | 317 | 317 | 317 |
| ***Panel B:*** Measuring EOHO by number of restaurants per capita | | | | | | |
| Post $\times$ log(EOHO restaurants per capita) | 0.006** | 0.008*** | 0.009*** | 0.011*** | 0.008*** | 0.008*** |
| | (0.003) | (0.003) | (0.003) | (0.003) | (0.002) | (0.002) |
| Mean DV | 0.096 | 0.096 | 0.096 | 0.096 | 0.096 | 0.096 |
| Observations | 88283 | 88283 | 88283 | 88283 | 88283 | 88283 |
| MSOA | 6791 | 6791 | 6791 | 6791 | 6791 | 6791 |
| Additional controls | 117 | 390 | 1209 | 4121 | 6929 | 9373 |
| Clusters | 317 | 317 | 317 | 317 | 317 | 317 |
| **Area by Week FE**: | NUTS1 | NUTS2 | NUTS3 | LAD | PCON | PCON x LAD |

**Notes:** Table presents difference-in-difference regression estimates studying the impact of the EOHO at the MSOA level on the emergence of new COVID19 infection clusters across the 13 calendar weeks from 24 to 36. The dependent variable is 1 in case an MSOA reported more than two new detected infections per calendar week. The independent variable in panel A measures the EOHO scheme as the log number of meals served in restaurants in an MSOA that participate in the EOHO scheme plus 1 divided by the population in the area. The independent variable in panel B measures the EOHO scheme as the log number of restaurants that participate in the EOHO scheme in an MSOA plus 1 divided by the population in the area. The specifications across panels explore the robustness to controlling for more granular non-linear time fixed effects. NUTS refers to the nomenclature unitÃ©s territoriales statistiques which subdivides the England into 11, 30 and 93 regions. LAD refers too local authority districts. PCON refers to Westminster parliamentary constituencies. Standard errors are clustered at the district level with starts indicating *** p< 0.01, ** p< 0.05, * p< 0.1.

Table A7: Reduced Form Impact of Rainfall on EOHO days on emergence of local COVID19 infection clusters: leads and lagged effects

| DV: Any new COVID19 cluster | (1) | (2) | (3) |
|---|---|---|---|
| Rainfall on EOHO days during lunch and dinner time | -0.011* | -0.012** | -0.013* |
| | (0.006) | (0.006) | (0.007) |
| Previous weeks Rainfall on EOHO days during lunch and dinner time | | -0.006 | -0.006 |
| | | (0.006) | (0.007) |
| Next weeks Rainfall on EOHO days during lunch and dinner time | | | -0.001 |
| | | | (0.007) |
| Mean DV | 0.143 | 0.143 | 0.143 |
| Observations | 33955 | 33955 | 33955 |
| Clusters | 317 | 317 | 317 |

**Notes:** Table presents difference-in-difference regression estimates studying the impact of the EOHO at the MSOA level on the emergence of new COVID19 infection clusters during calendar weeks 32 to 36. All regressions also control for local authority by week fixed effects. Standard errors are clustered at the district level with starts indicating *** $p < 0.01$, ** $p < 0.05$, * $p < 0.1$.

Table A8: Reduced Form Impact of Rainfall on EOHO days on emergence of local COVID19 infection clusters later in the week: alternative rainfall measures

| | (1) | (2) | (3) |
|---|---|---|---|
| ***Panel A:*** Any significant rainfall | | | |
| Significant Rainfall on EOHO days during lunch and dinner time | -0.027*** | -0.027*** | -0.027*** |
| | (0.009) | (0.009) | (0.009) |
| Significant Rainfall on Non-EOHO days during lunch and dinner time | | 0.001 | |
| | | (0.010) | |
| Significant Rainfall on EOHO days outside lunch and dinner hours | | | -0.008 |
| | | | (0.013) |
| Mean DV | 0.143 | 0.143 | 0.143 |
| Observations | 33955 | 33955 | 33955 |
| Clusters | 317 | 317 | 317 |
| ***Panel B:*** Rainfall in levels | | | |
| Rainfall on EOHO days during lunch and dinner time | -0.004* | -0.004* | -0.004* |
| | (0.002) | (0.002) | (0.002) |
| Rainfall on Non-EOHO days during lunch and dinner time | | 0.002 | |
| | | (0.002) | |
| Rainfall on EOHO days outside lunch and dinner hours | | | 0.000 |
| | | | (0.001) |
| Mean DV | 0.143 | 0.143 | 0.143 |
| Observations | 33955 | 33955 | 33955 |
| Clusters | 317 | 317 | 317 |

Notes: Table presents difference-in-difference regression estimates studying the impact of the EOHO at the MSOA level on the emergence of new COVID19 infection clusters during calendar weeks 32 to 36. All regressions also control for local authority by week fixed effects. Standard errors are clustered at the district level with starts indicating *** p< 0.01, ** p< 0.05, * p< 0.1.

Table A9: Reduced Form Impact of Rainfall on EOHO days on emergence of local COVID19 infection clusters later in the week: alternative dependent variables

| | (1) | (2) | (3) |
|---|---|---|---|
| ***Panel A:*** Any new COVID19 infection cluster | | | |
| Significant Rainfall on EOHO days during lunch and dinner time | -0.027*** | -0.027*** | -0.027*** |
| | (0.009) | (0.009) | (0.009) |
| Significant Rainfall on Non-EOHO days during lunch and dinner time | | 0.001 | |
| | | (0.010) | |
| Significant Rainfall on EOHO days outside lunch and dinner hours | | | -0.008 |
| | | | (0.013) |
| Mean DV | 0.143 | 0.143 | 0.143 |
| Observations | 33955 | 33955 | 33955 |
| Clusters | 317 | 317 | 317 |
| ***Panel B:*** log(# of new COVID19 cases+1) | | | |
| Significant Rainfall on EOHO days during lunch and dinner time | -0.044** | -0.044** | -0.042** |
| | (0.018) | (0.018) | (0.017) |
| Significant Rainfall on Non-EOHO days during lunch and dinner time | | -0.005 | |
| | | (0.016) | |
| Significant Rainfall on EOHO days outside lunch and dinner hours | | | -0.033 |
| | | | (0.023) |
| Mean DV | 0.249 | 0.249 | 0.249 |
| Observations | 33955 | 33955 | 33955 |
| Clusters | 317 | 317 | 317 |
| ***Panel C:*** # of new COVID19 cases | | | |
| Significant Rainfall on EOHO days during lunch and dinner time | -0.148** | -0.148** | -0.133* |
| | (0.074) | (0.074) | (0.071) |
| Significant Rainfall on Non-EOHO days during lunch and dinner time | | -0.072 | |
| | | (0.054) | |
| Significant Rainfall on EOHO days outside lunch and dinner hours | | | -0.295** |
| | | | (0.149) |
| Mean DV | 0.763 | 0.763 | 0.763 |
| Observations | 33955 | 33955 | 33955 |
| Clusters | 317 | 317 | 317 |

Notes: Table presents difference-in-difference regression estimates studying the impact of the EOHO at the MSOA level on the emergence of new COVID19 infection clusters during calendar weeks 32 to 36. All regressions also control for local authority by week fixed effects. Standard errors are clustered at the district level with starts indicating *** p< 0.01, ** p< 0.05, * p< 0.1.

Table A10: Reduced Form Impact of Rainfall on EOHO days on Google mobility across districts over time

| | (1) | (2) | (3) |
|---|---|---|---|
| ***Panel A:*** Recreation and Retail | | | |
| Significant Rainfall on EOHO days during lunch and dinner time | -1.668*** | | -1.549*** |
| | (0.341) | | (0.342) |
| Significant Rainfall on non-EOHO days during lunch and dinner time | | -0.707** | |
| | | (0.335) | |
| Significant Rainfall on EOHO days outside lunch and dinner hours | | | -0.373 |
| | | | (0.381) |
| Mean DV | -8.244 | -21.968 | -8.244 |
| Observations | 2401 | 4233 | 2401 |
| Clusters | 311 | 312 | 311 |
| ***Panel B:*** Grocery | | | |
| Significant Rainfall on EOHO days during lunch and dinner time | -0.823*** | | -0.681*** |
| | (0.220) | | (0.215) |
| Significant Rainfall on non-EOHO days during lunch and dinner time | | -0.625** | |
| | | (0.258) | |
| Significant Rainfall on EOHO days outside lunch and dinner hours | | | -0.470* |
| | | | (0.248) |
| Mean DV | -12.288 | -12.136 | -12.288 |
| Observations | 2893 | 4336 | 2893 |
| Clusters | 311 | 311 | 311 |
| ***Panel C:*** Parks | | | |
| Significant Rainfall on EOHO days during lunch and dinner time | -21.561*** | | -17.465*** |
| | (3.498) | | (3.305) |
| Significant Rainfall on non-EOHO days during lunch and dinner time | | -7.747*** | |
| | | (2.923) | |
| Significant Rainfall on EOHO days outside lunch and dinner hours | | | -8.881** |
| | | | (4.368) |
| Mean DV | 92.791 | 75.969 | 92.791 |
| Observations | 937 | 2179 | 937 |
| Clusters | 239 | 295 | 239 |
| ***Panel D:*** Transit | | | |
| Significant Rainfall on EOHO days during lunch and dinner time | -1.125** | | -0.762* |
| | (0.453) | | (0.437) |
| Significant Rainfall on non-EOHO days during lunch and dinner time | | 0.263 | |
| | | (0.503) | |
| Significant Rainfall on EOHO days outside lunch and dinner hours | | | -1.165** |
| | | | (0.558) |
| Mean DV | -35.748 | -27.783 | -35.748 |
| Observations | 2934 | 3947 | 2934 |
| Clusters | 311 | 311 | 311 |
| ***Panel D:*** Workplace | | | |
| Significant Rainfall on EOHO days during lunch and dinner time | -0.177** | | -0.119 |
| | (0.087) | | (0.088) |
| Significant Rainfall on non-EOHO days during lunch and dinner time | | -1.065*** | |
| | | (0.282) | |
| Significant Rainfall on EOHO days outside lunch and dinner hours | | | -0.195* |
| | | | (0.102) |
| Mean DV | -46.681 | -32.225 | -46.681 |
| Observations | 4624 | 4837 | 4624 |
| Clusters | 312 | 312 | 312 |
| ***Panel D:*** Residential | | | |
| Significant Rainfall on EOHO days during lunch and dinner time | 0.352*** | | 0.309*** |
| | (0.040) | | (0.041) |
| Significant Rainfall on non-EOHO days during lunch and dinner time | | 0.726*** | |
| | | (0.076) | |
| Significant Rainfall on EOHO days outside lunch and dinner hours | | | 0.150*** |
| | | | (0.050) |
| Mean DV | 11.417 | 7.504 | 11.417 |
| Observations | 4647 | 5533 | 4647 |
| Clusters | 311 | 311 | 311 |

Notes: Table presents difference-in-difference regression estimates studying the impact of the EOHO at the MSOA level on the emergence of new COVID19 infection clusters during calendar weeks 32 to 36. All regressions also control for local authority by week fixed effects. Standard errors are clustered at the district level with starts indicating *** p< 0.01, ** p< 0.05, * p< 0.1.

Table A11: Reduced Form Impact of Rainfall on EOHO days on Google mobility scores proxying visits to restaurants and cafe's across districts over time

| DV: Google mobility visits to restaurants | (1) | (2) | (3) |
|---|---|---|---|
| ***Panel A:*** Data window covering exactly the EOHO scheme | | | |
| Significant Rainfall on EOHO days during lunch and dinner time | -1.031** | | -0.964** |
| | (0.405) | | (0.390) |
| Significant Rainfall on non-EOHO days during lunch and dinner time | | -0.493 | |
| | | (0.427) | |
| Significant Rainfall on EOHO days outside lunch and dinner hours | | | -0.329 |
| | | | (0.523) |
| Mean DV | -8.244 | -21.968 | -8.244 |
| Observations | 2401 | 4233 | 2401 |
| Clusters | 311 | 312 | 311 |
| ***Panel B:*** Data window four week window prior to EOHO scheme | | | |
| Significant Rainfall on EOHO days during lunch and dinner time | 0.482 | | -0.021 |
| | (1.151) | | (1.136) |
| Significant Rainfall on non-EOHO days during lunch and dinner time | | 2.040 | |
| | | (1.408) | |
| Significant Rainfall on EOHO days outside lunch and dinner hours | | | 2.132** |
| | | | (0.932) |
| Mean DV | -21.407 | -28.633 | -21.407 |
| Observations | 3732 | 4979 | 3732 |
| Clusters | 311 | 312 | 311 |
| ***Panel C:*** Data window four weeks after the EOHO scheme | | | |
| Significant Rainfall on EOHO days during lunch and dinner time | 0.700 | | 0.696 |
| | (0.728) | | (0.725) |
| Significant Rainfall on non-EOHO days during lunch and dinner time | | -1.379* | |
| | | (0.764) | |
| Significant Rainfall on EOHO days outside lunch and dinner hours | | | -0.106 |
| | | | (0.759) |
| Mean DV | -13.965 | -18.172 | -13.965 |
| Observations | 2918 | 4478 | 2918 |
| Clusters | 311 | 312 | 311 |

**Notes:** Table presents regression estimates studying the impact of inclement weather on Google mobility proxies capturing visits to Restaurants and Cafes within local authority districts over time. Column (1) and (3) exploit intra-day variation in rainfall falling during core lunch and dinner hours and outside these hours to study its impact on mobility to restaurants on Mondays to Wednesdays during which the EOHO scheme would have been available during calendar weeks 32 to 36. Column (2) explores the impact of rainfall falling during core lunch and dinner hours on restaurant visits occurring from Thursdays to Sundays – days during which the EOHO discount would not have been available. Panel A focuses on the calendar weeks 32 to 36 when the EOHO was available, while Panel B and Panel C can be thought of as placebo exercises studying the rainfall and mobility relationships during times when the EOHO scheme was not available. All regressions control for district fixed effects and NUTS2 area by date fixed effects. Standard errors are clustered at the district level with starts indicating *** $p < 0.01$, ** $p < 0.05$, * $p < 0.1$.

Table A12: Quantification of EOHO Impact on Case Counts

| | Mid | Lower | Upper |
|---|---|---|---|
| ***Panel A:*** Overall cases during EOHO calendar weeks 32 to 36 | | | |
| ONS Infection Survey Estimate | 107,100 | 73,400 | 149,100 |
| Lab Confirmed COVID19 Cases | 45,382 | | |
| ***Panel B:*** % of infections due to EOHO | 0.116 | 0.106 | 0.146 |
| **Estimates of absolute case counts caused by EOHO** | | | |
| ***Panel C:*** direct infection counts | | | |
| based on lab confirmed COVID19 cases | 5,264 | 4,798 | 6,643 |
| based on ONS Infection Survey Estimate | 12,423 | 11,352 | 15,804 |
| based on ONS Infection Survey Lower | 8,514 | 7,759 | 10,744 |
| based on ONS Infection Survey Upper | 17,296 | 15,762 | 21,824 |
| ***Panel D:*** including indirect assuming 0.5 onward infections | | | |
| based on lab confirmed COVID19 cases | 16,646 | 16,179 | 18,024 |
| based on ONS Infection Survey Estimate | 39,283 | 35,800 | 49,569 |
| based on ONS Infection Survey Lower | 26,922 | 24,535 | 33,972 |
| based on ONS Infection Survey Upper | 54,689 | 49,839 | 69,008 |

**Notes:** Panel A presents the infection estimates for England between calendar weeks 32 to 36 inclusive. The table distinguishes between lab confirmed COVID19 infections and estimates derived from the representative ONS Infection Survey. Panel B provides the point estimates derived from the analysis expressed as the % of cases attributable to EOHO. Panel C provides absolute number of cases estimates of direct infections. Panel D provides an illustrative calculation of the dynamic impact of EOHO beyond week 36 assuming that each infection triggered 0.5 onward infections in the subsequent four week period.
